# PPARα is essential for retinal lipid metabolism and neuronal survival

**DOI:** 10.1186/s12915-017-0451-x

**Published:** 2017-11-28

**Authors:** Elizabeth A. Pearsall, Rui Cheng, Kelu Zhou, Yusuke Takahashi, H. Greg Matlock, Shraddha S. Vadvalkar, Younghwa Shin, Thomas W. Fredrick, Marin L. Gantner, Steven Meng, Zhongjie Fu, Yan Gong, Michael Kinter, Kenneth M. Humphries, Luke I. Szweda, Lois E. H. Smith, Jian-xing Ma

**Affiliations:** 1 Department of Ophthalmology, Boston Children’s Hospital, Harvard Medical School, Boston, MA 02115 USA; 2 Department of Physiology, University of Oklahoma Health Sciences Center, 941 Stanton L. Young Blvd., BSEB 328B, Oklahoma City, OK 73104 USA; 3 Section of Diabetes and Endocrinology, Department of Medicine, University of Oklahoma Health Sciences Center, Oklahoma City, OK 73104 USA; 4 Aging and Metabolism Research Program, Oklahoma Medical Research Foundation, Oklahoma City, OK 73104 USA; 5 The Lowy Medical Research Institute, La Jolla, CA 92037 USA

**Keywords:** Age-related macular degeneration, Retina, Neurodegeneration, Retinal energy metabolism, Mitochondria, Lipids, Fatty acid oxidation

## Abstract

**Background:**

Peroxisome proliferator activated receptor-alpha (PPARα) is a ubiquitously expressed nuclear receptor. The role of endogenous PPARα in retinal neuronal homeostasis is unknown. Retinal photoreceptors are the highest energy-consuming cells in the body, requiring abundant energy substrates. PPARα is a known regulator of lipid metabolism, and we hypothesized that it may regulate lipid use for oxidative phosphorylation in energetically demanding retinal neurons.

**Results:**

We found that endogenous PPARα is essential for the maintenance and survival of retinal neurons, with *Pparα* ^*-/-*^ mice developing retinal degeneration first detected at 8 weeks of age. Using extracellular flux analysis, we identified that PPARα mediates retinal utilization of lipids as an energy substrate, and that ablation of PPARα ultimately results in retinal bioenergetic deficiency and neurodegeneration. This may be due to PPARα regulation of lipid transporters, which facilitate the internalization of fatty acids into cell membranes and mitochondria for oxidation and ATP production.

**Conclusion:**

We identify an endogenous role for PPARα in retinal neuronal survival and lipid metabolism, and furthermore underscore the importance of fatty acid oxidation in photoreceptor survival. We also suggest PPARα as a putative therapeutic target for age-related macular degeneration, which may be due in part to decreased mitochondrial efficiency and subsequent energetic deficits.

**Electronic supplementary material:**

The online version of this article (doi:10.1186/s12915-017-0451-x) contains supplementary material, which is available to authorized users.

## Background

In order to transduce light signals, photoreceptors must maintain a steep membrane gradient that constantly fluctuates in response to light stimuli, which is energetically demanding [1]. Photoreceptor mitochondria therefore function at full capacity with a limited respiratory reserve, and require abundant energy substrates and oxygen [2]. Fuel shortages or impairment of mitochondrial function are therefore detrimental to photoreceptor survival, and quickly result in neurodegeneration. Accordingly, mitochondrial dysfunction has been linked to a litany of retinal diseases, including age-related macular degeneration, diabetic retinopathy, macular telangtasia and, potentially, retinal degenerations [3]. Further, a growing body of evidence suggests that neurodegenerative diseases of the brain and peripheral nervous system may also have a metabolic root [4, 5]. It is therefore imperative to further the understanding of neuronal energy metabolism such that additional therapeutics targeting metabolic dysregulation can be developed.

Age-related macular degeneration (AMD) is characterized by degeneration of the neural retina and retinal pigment epithelium with subsequent compensatory but ultimately pathological neovascularization in advanced stages. This response may be due in part to mitochondrial dysfunction with aging and subsequent decreased ATP production in photoreceptors [3, 6]. Previous dogma suggested that retinal neurons rely exclusively on glucose as a fuel source [1], but a recent study identified that retinal neurons use both glucose and lipids for ATP production [7]. However, because lipid use in retinal neurons is newly identified, the mediators of retinal fatty acid oxidation (FAO) are currently unknown. Here we identify a central role for peroxisome proliferator activated receptor-alpha (PPARα) in retinal FAO, and furthermore demonstrate that FAO is crucial for the maintenance and survival of retinal neurons in physiological conditions.

Although PPARα activation has therapeutic effects in neurodegenerative conditions [8], its endogenous role in neuronal homeostasis and survival is incompletely understood. PPARα is a regulator of FAO, activating transcription of mitochondrial and cell membrane lipid transporters as well as FAO enzymes [9]. We find that *Pparα* ^*-/-*^ retinas have impaired use of lipids as an oxidizable substrate for ATP production, which may be due to changes in the transcriptional regulation of mitochondrial lipid transporters important for FAO and/or mitochondrial FAO enzymes. To compensate for decreased lipid use, *Pparα* ^*-/-*^ retinas use more glycolytic products for oxidative phosphorylation, but ultimately develop bioenergetic insufficiency and subsequent neurodegeneration.

Lipids and glucose are both important retinal energy substrates. Lipid oxidation in peroxisomes and mitochondria occurs through FAO, yielding acetyl-CoA for the citric acid cycle and reducing equivalents for the electron transport chain. Glucose is catabolized to pyruvate through glycolysis, which is then converted to lactate, or to acetyl-CoA for the citric acid cycle. Recent studies have suggested that lipids are used for oxidative phosphorylation by retinal neurons, while most glucose is converted to lactate through aerobic glycolysis [7]. However, the regulation of FAO and preferential, context-dependent use of each substrate remain incompletely understood. Our findings also suggest that lipids are used for oxidative phosphorylation in normal conditions and that pyruvate may be more readily used as an oxidizable substrate under the stress of impaired FAO, such as in *Pparα* ^*-/-*^ retinas. These findings further underscore the importance of lipids as oxidizable substrates for ATP production in retinal neurons, and contribute to the basic understanding of retinal energy metabolism.

Together, these findings identify a central role for PPARα in lipid metabolism and retinal neuronal survival, and further delineate retinal metabolic flux. They also suggest PPARα as a new therapeutic target for neurodegenerative diseases of metabolic origin, including AMD, a leading cause of blindness in the elderly [10], and potentially in retinal degenerations [11].

## Results

### Essential role of endogenous PPARα in neuronal survival

**Fig. 1:**
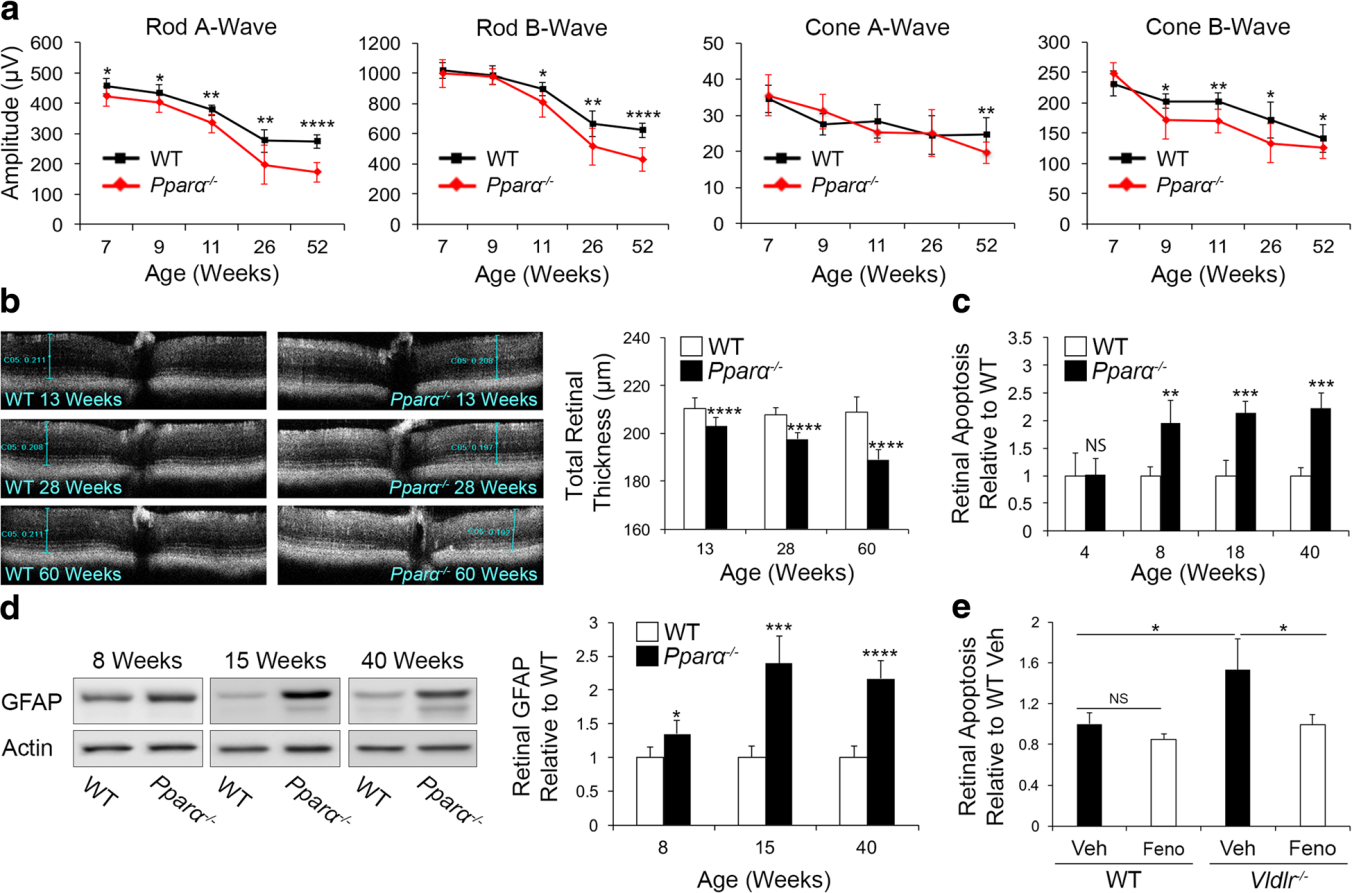
Essential role of PPARα in retinal neuronal survival. **a** Longitudinal electroretinography revealed that retinal function (rod and cone A-waves and B-waves) declined in *Pparα* ^*-/-*^ mice relative to wild-type (WT) starting at 7 weeks (7, 9, 11, and 26 weeks, n = 5 mice/genotype; 52 weeks, n = 8 mice/genotype). **b** Cross-sectional optical coherence tomography demonstrated that total retinal thickness progressively declined in *Pparα* ^*-/-*^ mice and remained constant in WT (13 weeks, n = 9 mice/genotype; 26 weeks, WT n = 9, *Pparα* ^*-/-*^ n = 8; 60 weeks, n = 8 mice/genotype). **c** Retinal cell death ELISA identified that total retinal apoptosis was significantly increased in *Pparα* ^*-/-*^ retinas relative to WT beginning at 8 weeks of age (n = 5 retinas/genotype per time point). **d** Retinal GFAP levels were increased in *Pparα* ^*-/-*^ retinas relative to WT, suggesting retinal gliosis (8, 15 weeks, n = 5 retinas/genotype; 40 weeks, n = 6 retinas/genotype). **e** Retinal cell death ELISA revealed that total retinal apoptosis was significantly increased in *Vdllr* ^*-/-*^ mice and restored to baseline by treatment with PPARα agonist fenofibric acid (Feno) (WT Veh, n = 7; WT Feno, n = 8; *Vdllr* ^*-/-*^ Veh, n = 7; *Vdllr* ^*-/-*^ Feno, n = 8). Data are expressed as mean ± SEM. *P ≤ 0.05; **P ≤ 0.01; ***P ≤ 0.001; ****P ≤ 0.0001, unpaired two-tailed Student’s *t* test (**a**–**d**) or one-way ANOVA with Tukey’s post-hoc comparison (**e**)

Serial electroretinogram (ERG) recordings demonstrated that retinal function declined in *Pparα* ^*-/-*^ mice relative to wild-type (WT) C57Bl6/J mice (Fig. 1a). The rod A-wave declined first and was the most significantly decreased, suggesting that rod photoreceptors were the most significantly affected cell type. The murine retina is rod-dominant [12] and PPARα is highly expressed in photoreceptors [7], so these findings were consistent with prior observations. Total retinal thickness declined progressively with age in *Pparα* ^*-/-*^ mice as demonstrated by optical coherence tomography (OCT) (Fig. 1b). Consistent with prior observations, ERG response declined in C57Bl6/J WT mice with age, while total retinal thickness remained unchanged (Fig. 1a, b), suggesting that the ERG decline in WT mice was unrelated to retinal degeneration, but rather might have been due to increased resistance of retinal neurons to electrical stimulus in WT mice, as suggested previously [13]. Total retinal apoptosis was unchanged at 4 weeks of age in *Pparα* ^*-/-*^ animals, but increased at 8, 18, and 40 weeks of age (Fig. 1c), which in combination with a progressive decline in retinal function and retinal thickness suggested persistent neurodegeneration in the absence of PPARα. We injected PPARα-expressing adenovirus intravitreally in 4-week-old *Pparα* ^*-/-*^ animals to selectively restore expression of PPARα in retinal neurons, and found that at 8 weeks of age, retinal apoptosis was significantly decreased in *Pparα* ^*-/-*^ animals injected with PPARα-expressing adenovirus relative to those injected with GFP-expressing adenovirus (Additional file 1: Figure S1F). This suggested that loss of PPARα in retinal neurons was operative in the observed neurodegenerative phenotype. Glial fibrillary acidic protein (GFAP) levels were increased in *Pparα* ^*-/-*^ retinas, suggesting retinal gliosis (Fig. 1d). Consistent with prior findings, we observed a 45 kDa isoform of GFAP after 15 weeks of age [14], which was also elevated in *Pparα* ^*-/-*^ retinas (Additional file 2: Figure S2D). In very low density lipoprotein receptor knockout (*Vldlr* ^*-/-*^) mice, a neurodegenerative neovascular model with some characteristics of neovascular AMD, including photoreceptor energy insufficiency [7, 15], treatment with PPARα agonist fenofibric acid decreased total retinal apoptosis (Fig. 1e). Taken together, these findings suggest that endogenous PPARα is essential for retinal neuronal survival under normal conditions, and that PPARα activation protects against retinal degeneration in an AMD-like model with photoreceptor energy deficits.

### Decreased vascular density in *Pparα*^*-/-*^ retinas

**Fig. 2:**
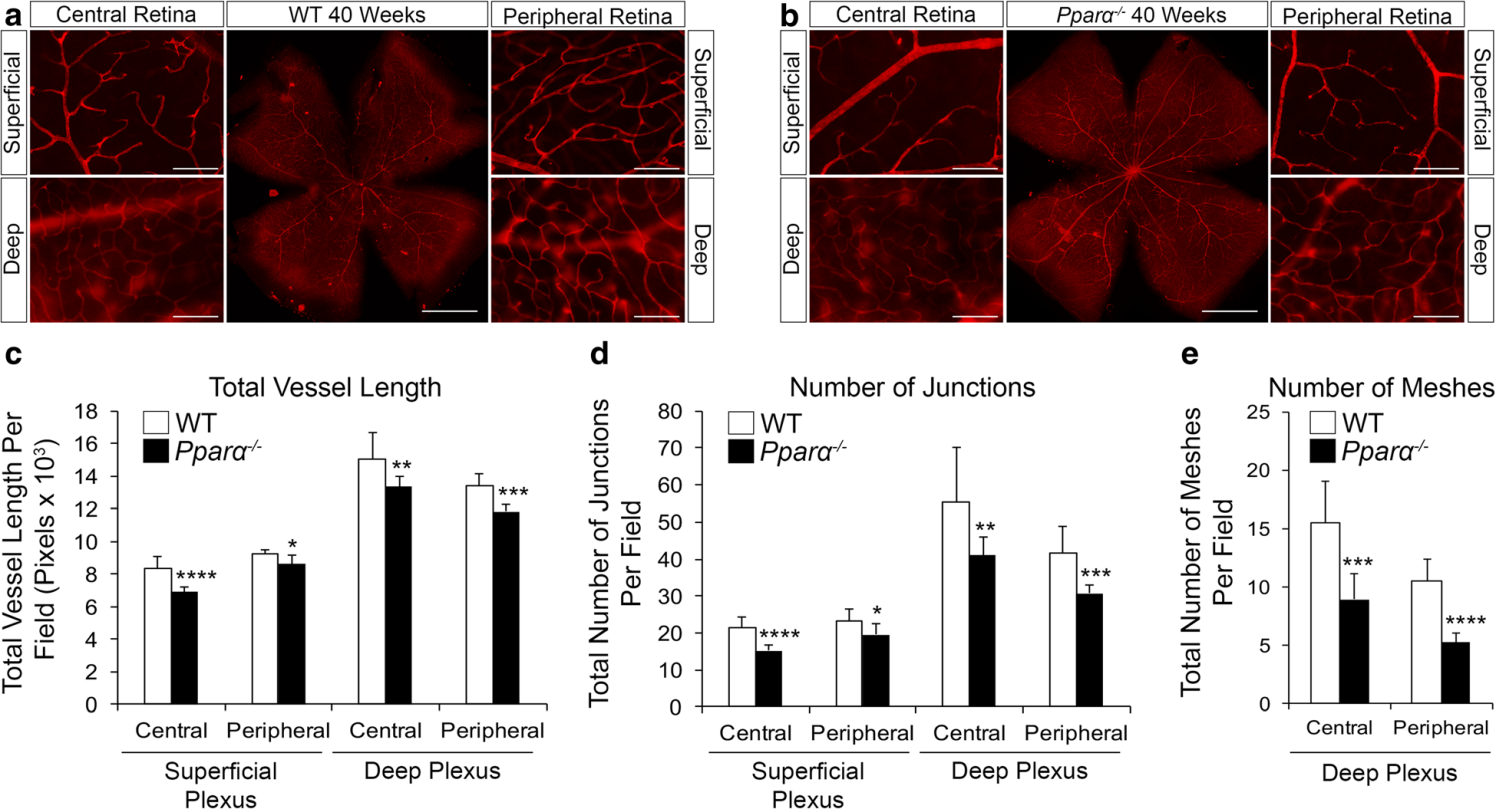
Decreased vascular density in *Pparα* ^*-/-*^ retinas. **a** Representative images of isolectin-labeled microvessels in retinal wholemount and single fields of superficial and deep vascular plexuses in central and peripheral retina of 40-week-old wild-type (WT) retinas. **b** Representative images of 40-week-old *Pparα* ^*-/-*^ retinas. **c** Decreased total vessel length in superficial and deep vascular plexuses of *Pparα* ^*-/-*^ retinas. **d** Decreased number of vascular junctions in superficial and deep vascular plexuses of *Pparα* ^*-/-*^ retinas. **e** Decreased number of meshes in deep vascular plexus of *Pparα* ^*-/-*^ retinas (adult retinas do not have meshing in the superficial plexus) (WT, n = 7; *Pparα* ^*-/-*^, n = 8 retinas). *P ≤ 0.05; **P ≤ 0.01; ***P ≤ 0.001; ****P ≤ 0.0001, Student’s *t* test

Energetically demanding photoreceptors dictate vascular outgrowth to ensure that metabolic needs are met [7]. At 40 weeks of age, vascular density in the superficial and deep vascular plexuses was decreased in *Pparα* ^*-/-*^ retinas, as demonstrated by decreased total vascular length (Fig. 2c), decreased number of vascular junctions (Fig. 2d), and decreased number of vascular meshes in the deep vascular plexus (Fig. 2e). We suggest that the decreased number and metabolic activity of retinal neurons may dictate the survival of retinal blood vessels in *Pparα* ^*-/-*^ retinas. Neurodegeneration is first detectable by 8 weeks of age in *Pparα* ^*-/-*^ retinas (Fig. 1c). Consistent with retinal neurons controlling vascular survival, vascular density was modestly decreased in 8-week-old *Pparα* ^*-/-*^ retinas, particularly in the central retina, which is the highest energy-consuming region of the retina (Additional file 3: Figure S3). Importantly, we have previously demonstrated that hypoxic signaling is not activated in *Pparα* ^*-/-*^ retinas under normal conditions [16], suggesting that the relatively subtle decreases of vascular density are not sufficient to induce neuronal ischemia. Other hallmarks of vascular dysfunction, such as vascular hyperpermeability, formation of acellular capillaries, and leukostasis, are also unchanged in *Pparα* ^*-/-*^ retinas under normal conditions [17]. We have also previously identified that PPARα is most highly expressed in photoreceptors [7]. Together, these findings suggest that the relatively subtle decreases in vascular density are unlikely to be operative in the robust neurodegenerative phenotype, but are rather likely secondary to decreased neuronal energetic demand due to decreased metabolic rates and loss of retinal neurons.

### Role of PPARα in retinal lipid metabolism

**Fig. 3:**
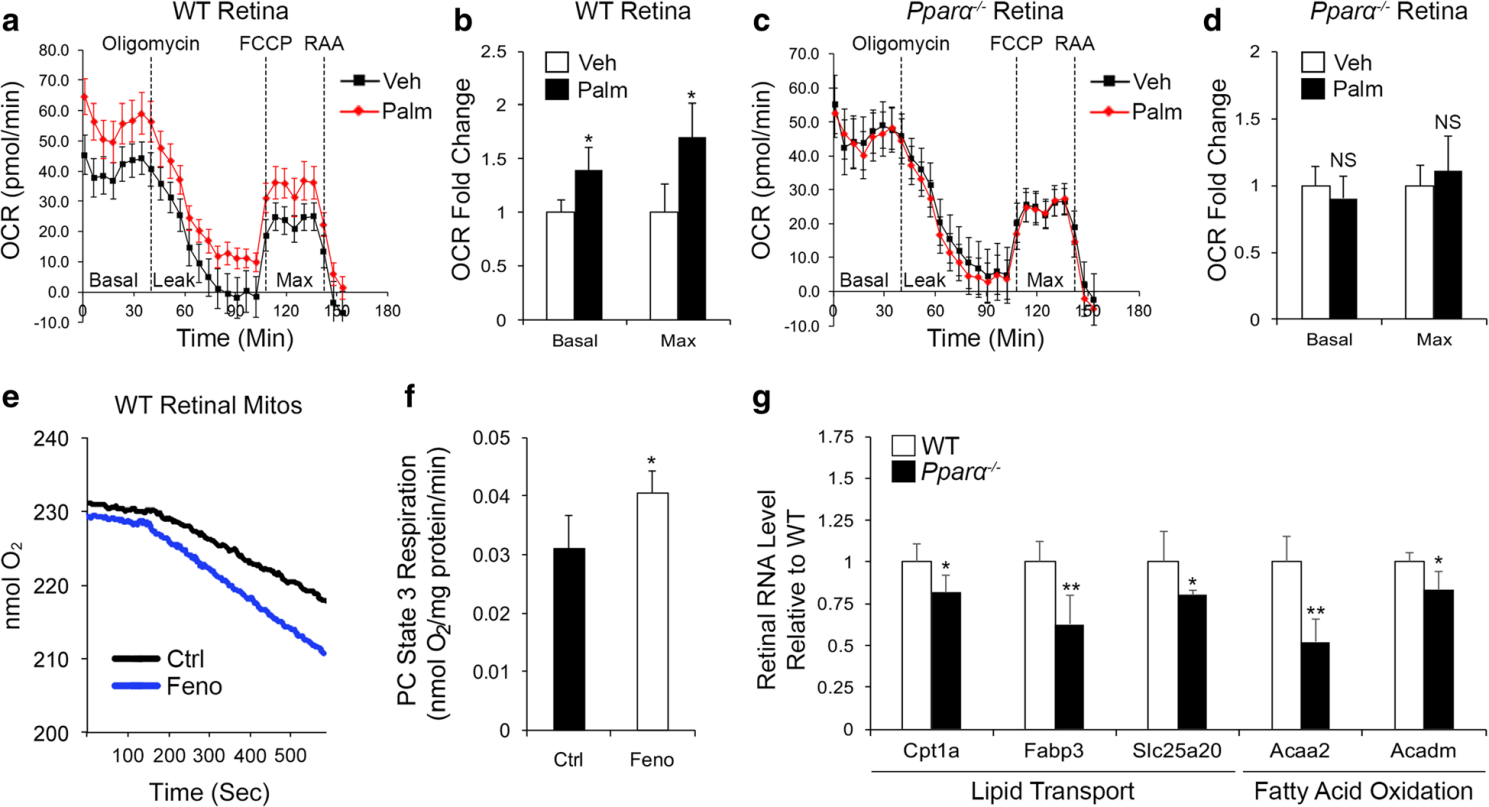
PPARα is essential for utilization of lipid for oxidative phosphorylation. **a**, **b** Measurement of (**a**) retinal oxygen consumption with a Seahorse XFe analyzer revealed that (**b**) baseline and maximal retinal oxygen consumption rate (OCR) were significantly increased in wild-type (WT) retinas when long-chain fatty acid palmitate (Palm) was provided, suggesting that Palm is used as an oxidizable substrate in normal conditions (BSA, n = 12; Palm, n = 11). **c**, **d** Retinal OCR was unaffected by Palm in *Pparα* ^*-/-*^ retinas, suggesting that retinal use of lipid as an oxidizable substrate is impaired in the absence of PPARα (BSA, n = 9; Palm, n = 12). **e**, **f** Oral administration of PPARα agonist fenofibrate (Feno) for 1 month prior to tissue harvest increased oxygen consumption in isolated rat retinal mitochondria (mitos) provided with lipid palmitoylcarnitine (PC) as an oxidizable substrate (n = 4). **g** RNA levels of lipid transporters and fatty acid oxidation (FAO) enzymes were decreased in *Pparα* ^*-/-*^ retinas (n = 5/genotype). *P ≤ 0.05; **P ≤ 0.01; ***P ≤ 0.001, unpaired two-tailed Student’s *t* test

These findings prompted us to explore the role of PPARα in retinal energy metabolism. Therefore, we first examined FAO in WT and *Pparα* ^*-/-*^ retinas. To ensure that any changes in substrate use were not secondary to neurodegeneration, we used 4-week-old animals, prior to the onset of neurodegeneration in *Pparα* ^*-/-*^ retinas (Fig. 1c). In WT retinas, fatty acid palmitate increased oxygen consumption rate (OCR) (Fig. 3a, b), which was reversed by the carnitine palmitoyltransferase 1A (Cpt1a) inhibitor etomoxir (Additional file 4: Figure S4A, B), suggesting that lipid is used for oxidative phosphorylation by WT retinas. In contrast, palmitate did not increase OCR in *Pparα* ^*-/-*^ retinas (Fig. 3c, d), which was not affected by etomoxir (Additional file 4: Figure S4C, D), suggesting that lipid is not used for oxidative phosphorylation in the absence of PPARα. Further, oral administration of the PPARα agonist fenofibrate for 4 weeks prior to tissue harvest increased oxygen consumption in isolated rat retinal mitochondria provided with lipid palmitoylcarnitine as an oxidizable substrate, suggesting that PPARα activation increased FAO (Fig. 3e, f). PPARα regulates FAO by activating transcription of lipid transporter and FAO target genes [9]. We measured RNA levels of a panel of lipid transporters and FAO enzymes in 4-week-old WT and *Pparα* ^*-/-*^ retinas, and found reductions in lipid transporter Cpt1A, which is considered the rate limiting enzyme for FAO, fatty acid binding protein 3 (Fabp3), and solute carrier 25a20 (Slc25a20) (Fig. 3g). RNA levels of most FAO enzymes were unaffected by PPARα ablation (Additional file 4: Figure S4E), with the exception of acetyl-CoA acyltransferase 2 (Acaa2) and medium-chain acetyl-CoA dehydrogenase (Acadm), which were decreased in *Pparα* ^*-/-*^ retinas (Fig. 3g). Taken together, these findings suggest that PPARα is essential for retinal utilization of lipids for ATP production, which is regulated by transcription of lipid-metabolizing PPARα target genes, including lipid transporters and FAO enzymes.

### Compensatory use of glycolytic products for oxidative phosphorylation in *Pparα*^*-/-*^ retinas

**Fig. 4:**
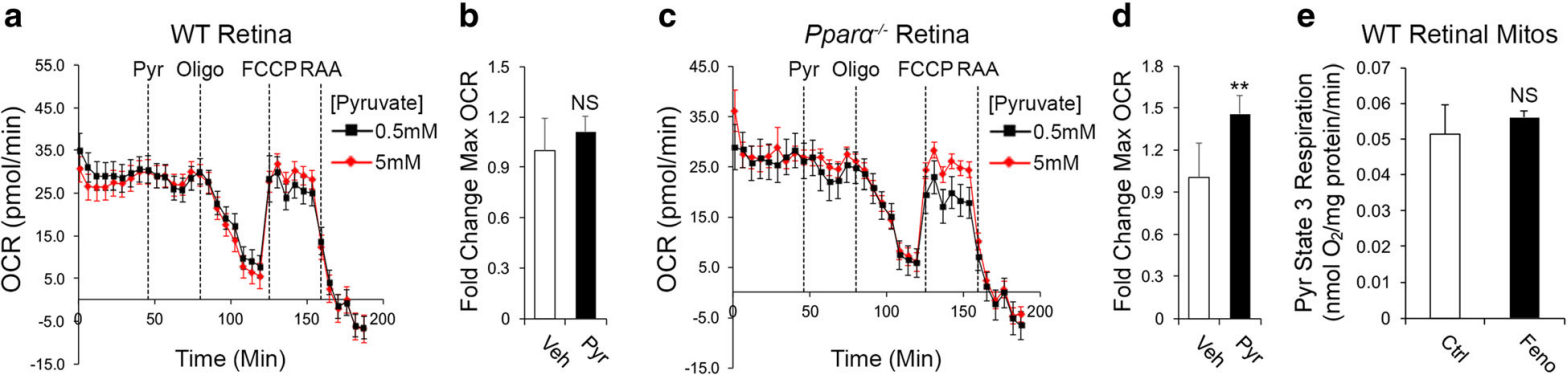
Compensatory use of glycolytic products for oxidative phosphorylation in the absence of PPARα. **a**, **b** In wild-type retinas, OCR was unchanged by addition of 5 mM pyruvate (Pyr) relative to 0.5 mM Pyr (Veh) control, suggesting that pyruvate was not readily used as an oxidizable substrate in normal conditions (Veh, n = 11; Pyruvate, n = 10). **c, d** In *Pparα* ^*-/-*^ retinas, maximal OCR was increased by pyruvate, suggesting that it was more readily used as an oxidizable substrate in the absence of PPARα (Veh, n = 11; Pyruvate, n = 9). **e** Oral administration of PPARα agonist Fenofibrate (Feno) prior to tissue harvest did not change oxygen consumption in isolated rat retinal mitochondria (Mitos) provided with pyruvate as an oxidizable substrate (n = 4). *P ≤ 0.05; **P ≤ 0.01; ****P ≤ 0.0001, unpaired two-tailed Student’s *t* test

To further explore PPARα’s role in retinal energy metabolism, we examined whether PPARα loss affected the use of glycolytic products for oxidative metabolism. Prior findings demonstrated that in normal conditions, the majority of glucose is converted to lactate through aerobic glycolysis in the retina [7]. We hypothesized that *Pparα* ^*-/-*^ retinas may compensate for impaired FAO by using more glycolytic products for oxidative phosphorylation, and thus measured oxygen consumption in response to pyruvate, the primary glycolytic product used to generate acetyl-CoA for aerobic respiration. In WT retinas, increasing pyruvate (5 mM vs. 0.5 mM) did not affect maximal OCR (Fig. 4a, b), supporting the notion that pyruvate is not readily used for oxidative phosphorylation in WT retinas [7]. In contrast, pyruvate significantly increased maximal OCR in *Pparα* ^*-/-*^ retinas (Fig. 4b, c), suggesting that glycolytic products are more readily used for oxidative phosphorylation in the absence of PPARα. Oral administration of the PPARα agonist fenofibrate in WT animals prior to tissue harvest did not affect OCR in isolated retinal mitochondria provided with pyruvate as an oxidizable substrate (Fig. 4e, Additional file 5: Figure S5), suggesting that increased use of pyruvate in *Pparα* ^*-/-*^ retinas is a compensatory effect, and is not directly regulated by PPARα.

### Impaired mitochondrial function in *Pparα*^*-/-*^ retinas

**Fig. 5:**
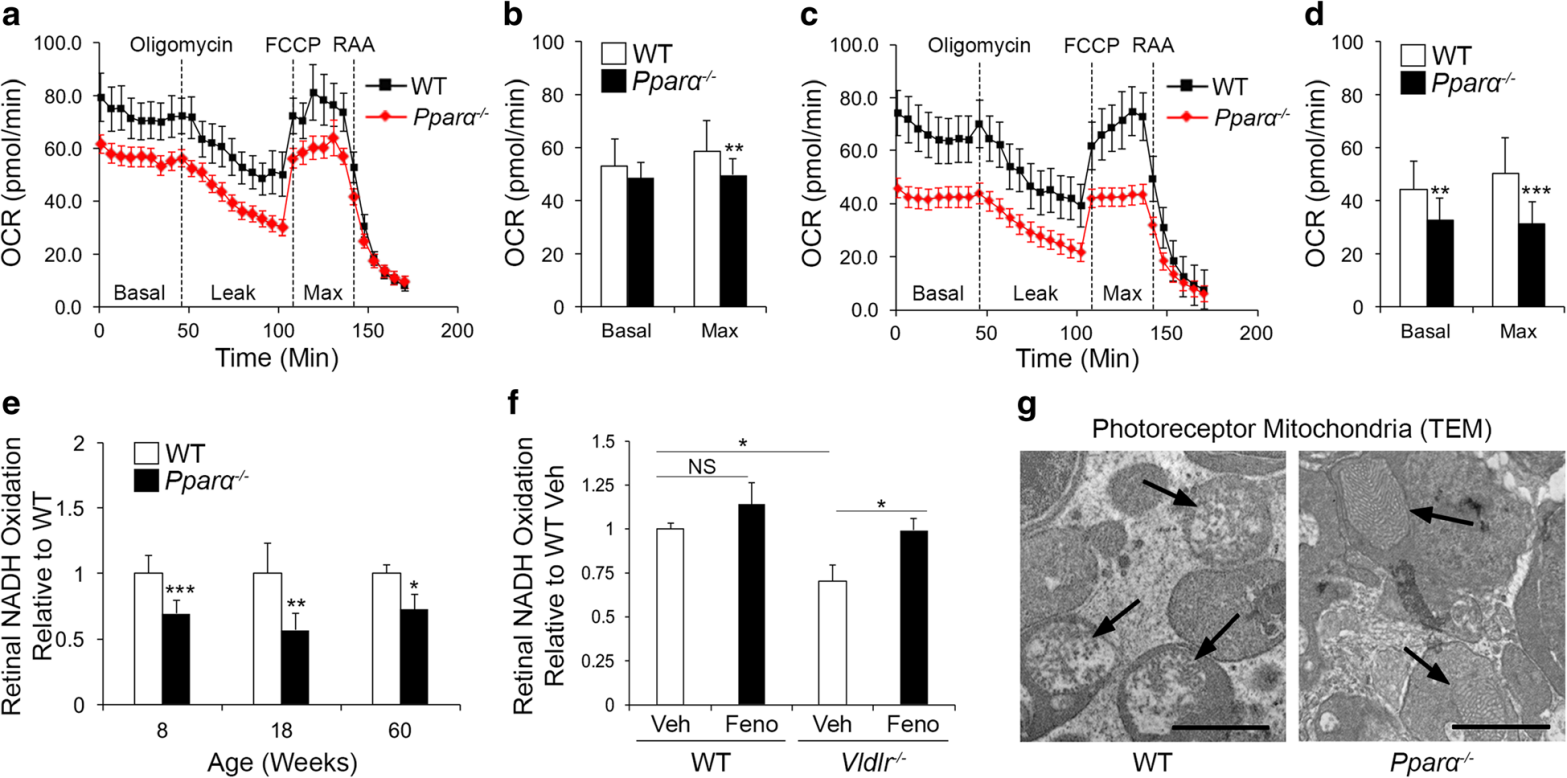
Diminished mitochondrial function in *Pparα* ^*-/-*^ retinas. **a**–**d** Basal and maximal mitochondrial oxygen consumption rate (OCR) were decreased in (**a**, **b**) 8-week-old and (**c**, **d**) 12-week-old *Pparα* ^*-/-*^ retinas relative to age-matched wild-type (WT) controls (WT 8 weeks, n = 17; *Pparα* ^*-/-*^ 8 weeks, n = 20; WT 12 weeks, n = 21; *Pparα* ^*-/-*^ 12 weeks, n = 15). **e** Retinal NADH oxidation was decreased in *Pparα* ^*-/-*^ retinas relative to age-matched WT controls beginning at 8 weeks of age (8 weeks WT, n = 10; *Pparα* ^*-/-*^, n = 9; 18 weeks, n = 6/genotype; 60 weeks, n = 3/genotype). **f** Retinal NADH oxidation was decreased in 11-week-old *Vldlr* ^*-/-*^ retinas and restored by treatment with PPARα agonist fenofibric acid (Feno) (WT Veh, n = 7; WT Feno-FA, n = 8; *Vdllr* ^*-/-*^ Veh, n = 7; *Vdllr* ^*-/-*^ Feno-FA, n = 8). **g** Transmission electron microscopy revealed that at 40 weeks of age, photoreceptor mitochondria had an “active” compressed morphology in WT retinas, and an “inactive” orthodox morphology in *Pparα* ^*-/-*^ retinas (n = 3/genotype). *P ≤ 0.05; **P ≤ 0.01; ***P ≤ 0.001; ****P ≤ 0.0001, unpaired two-tailed Student’s *t* test (**a**–**e**) or one-way ANOVA with Tukey’s post-hoc comparison (**f**)

We next characterized retinal mitochondrial activity in *Pparα* ^*-/-*^ mice after the onset of retinal degeneration. At 8 weeks of age, basal and maximal retinal OCR were significantly decreased *Pparα* ^*-/-*^ retinas (Fig. 5a, b), and further declined by 12 weeks of age (Fig. 5c, d). This was partially restored by intravitreal injection of PPARα-expressing adenovirus (Additional file 6: Figure S6A, B). Further, siRNA knockdown of PPARα significantly decreased mitochondrial respiration in cultured R28 rod precursor cells (Additional file 6: Figure S6C, D). Oxidation of exogenous NADH, reflective of electron transport chain activity, was decreased in *Pparα* ^*-/-*^ retinas relative to WT (Fig. 5e). Addition of electron transport chain inhibitor rotenone to the assay inhibited NADH oxidation by more than 10-fold and normalized NADH oxidation in *Pparα* ^*-/-*^ retinas relative to WT (Additional file 6: Figure S6E). Further, in *Vldlr* ^*-/-*^ retinas, NADH oxidation was similarly decreased, and restored by systemic treatment with PPARα agonist fenofibric acid (Fig. 5f), suggesting that PPARα activation increased retinal energy efficiency in an AMD-like model. Transmission electron microscopy (TEM) revealed that photoreceptor mitochondrial morphology in *Pparα* ^*-/-*^ retinas was consistent with mitochondria in the orthodox (less active) state relative to WT mitochondria, which displayed an active, compressed morphology [18], as supported by our enzymatic analysis (Fig. 5g). However, we subsequently identified that electron transport chain and Krebs cycle proteins were unchanged in *Pparα* ^*-/-*^ retinas (Additional file 7: Figure S7D, E), suggesting that the abundance of retinal mitochondria was unlikely to be changed, and that the observed functional and morphologic changes were likely due to the established deficits in substrate use.

### Proteomic analysis of *Pparα*^*-/-*^ retinas

**Fig. 6:**
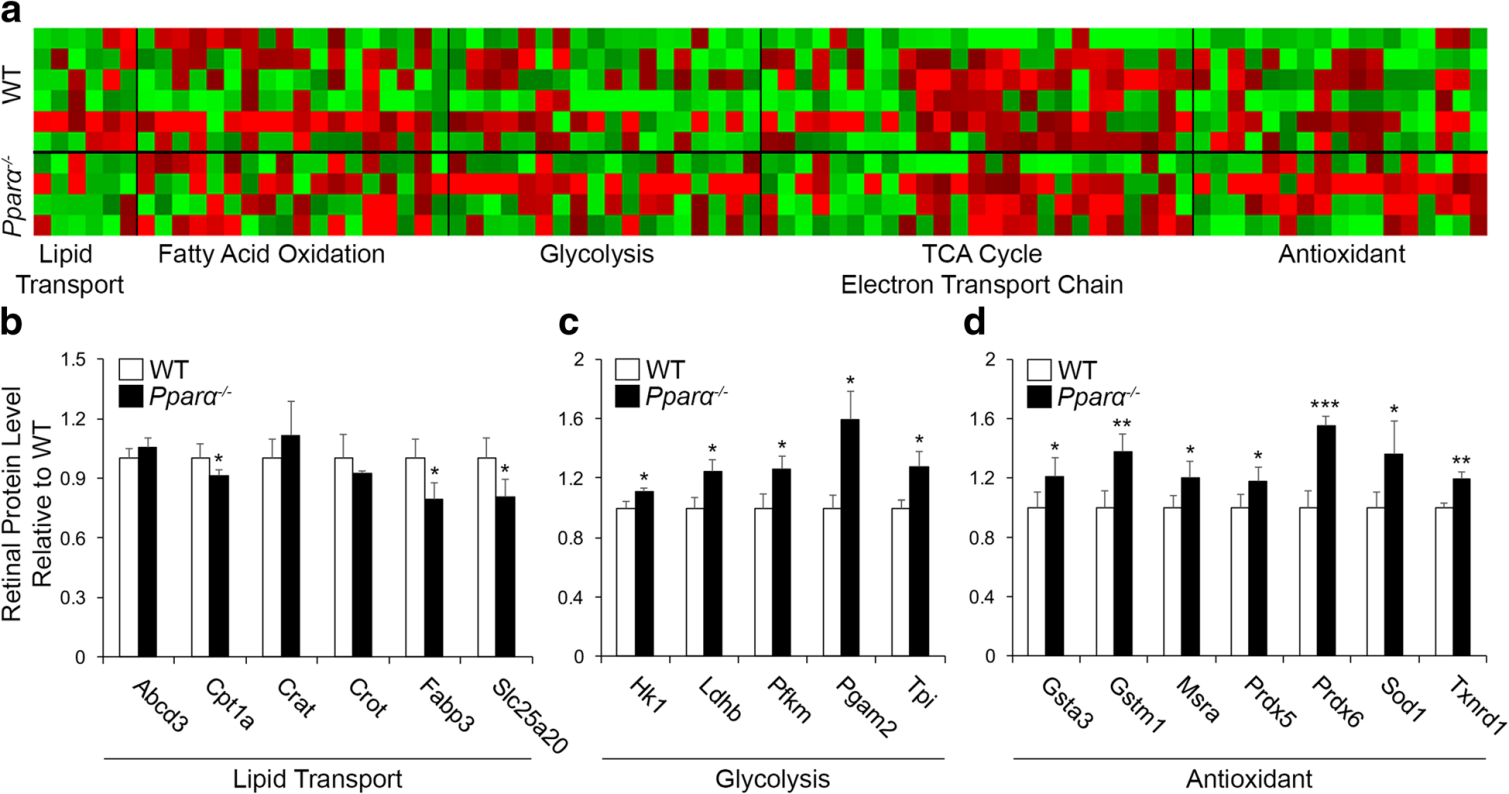
Proteomic analysis of *Pparα* ^*-/-*^ retinas. **a** Heatmap representation of lipid transporters, fatty acid oxidation, glycolytic, citric acid cycle, electron transport chain, and antioxidant enzymes measured by proteomic analysis. Please refer to Additional file 7: Figure S7 for full analysis. **b** Lipid transporters ATP binding cassette subfamily d member 3 (Abcd3), carnitine acetyl-transferase (Crat), and carnitine o-octanyltransferase (Crot) were unchanged in 40-week-old *Pparα* ^*-/-*^ retinas relative to age-matched wild-type (WT). Carnitine palmitoyltransferase 1a (Cpt1a), fatty acid binding protein (Fabp3), and solute carrier family 25 member 20 (Slc25a20) were decreased in *Pparα* ^*-/-*^ retinas. **c** Glycolytic enzymes hexokinase 1 (Hk1), lactate dehydrogenase-brain (Ldhb), phosphofructokinase muscle (Pfkm), phosphoglycerate mutase 2 (Pgam2), and triosephosphate isomerase 1 (Tpi) were increased in *Pparα* ^*-/-*^ retinas. **d** Antioxidant enzymes glutathione s-transferase alpha-3 (Gsta3), glutathione s-transferase mu 1 (Gstm1), peroxidase-5 and -6 (Prdx5, Prdx6), and thioredoxin reductase 1 (Txnrd1) were increased in *Pparα* ^*-/-*^ retinas (WT, n = 6; *Pparα* ^*-/-*^, n = 4). *P ≤ 0.05, **P ≤ 0.01, ***P ≤ 0.001, unpaired two-tailed Student’s *t* test

To take an unbiased approach towards identifying metabolic pathways responsible for PPARα regulation of retinal energy metabolism, we utilized a multiplexed quantitative proteomics mass spectrometry method to measure retinal levels of lipid transporters and enzymes essential for FAO, glycolysis, the citric acid cycle, the electron transport chain, and antioxidant enzymes [19] in *Pparα* ^*-/-*^ retinas at 40 weeks of age. Consistent with earlier RNA analysis, lipid transporters Cpt1a, Fapb3, and Slc25a20 were significantly decreased in *Pparα* ^*-/-*^ retinas (Fig. 6b). Glycolytic enzymes were up-regulated in *Pparα* ^*-/-*^ retinas (Fig. 6c, Additional file 7: Figure S7C), which may partially compensate for the inability to utilize lipid as a fuel source. Most FAO enzymes were unchanged in *Pparα* ^*-/-*^ retinas, with the exception of hydroxy-CoA dehydrogenase (Hadh), which was up-regulated by PPARα ablation, potentially also as a compensatory response to decreased overall lipid use (Additional file 7: Figure S7B). Krebs cycle and electron transport chain enzymes were unaffected by Pparα ablation (Additional file 7: Figure S7D, E). We observed that antioxidant enzymes were increased in *Pparα* ^*-/-*^ retinas (Fig. 6d, Additional file 7: Figure S7F), suggesting increased reactive oxygen species generation, potentially as a result of decreased mitochondrial efficiency. However, the most significantly upregulated antioxidant enzymes at 40 weeks of age were unchanged in 4-week-old *Pparα* ^*-/-*^ retinas (Additional file 8: Figure S8), suggesting that oxidative stress may not be responsible for neurodegeneration but is rather a byproduct of prolonged mitochondrial dysfunction occurring after the onset of neurodegeneration.

## Discussion

The retina has high metabolic demands requiring abundant energy substrates, and the ability to utilize lipids as a fuel source may confer a survival advantage. Lipids yield more ATP than glucose when used as an oxidizable substrate [20]. Lipid use may also support continued ATP production during hypoglycemic fasting periods, when fatty acids released from adipose tissue can be used in lieu of glucose for energy production [21].

Interestingly, our recent study identified that in the retina, the majority of glucose is converted to lactate through aerobic glycolysis, and that lipids are the predominant substrate for oxidative phosphorylation [7]. This is supported by our current findings, which demonstrate that WT retinas readily use lipids for oxidative phosphorylation, and that pyruvate, the primary glycolytic product used as a substrate for oxidative phosphorylation, did not affect mitochondrial respiration. In contrast, use of lipids for oxidative phosphorylation was impaired in *Pparα* ^*-/-*^ retinas, while pyruvate was more readily used, potentially in a compensatory effect. The relative roles of glucose versus lipid metabolism in the retina and preferential, context-dependent use of each substrate is incompletely understood. In this study, we underscore the importance of lipids for oxidative metabolism, finding that impaired lipid use results in the compensatory use of pyruvate, and ultimately in retinal energy insufficiency and subsequent neurodegeneration. This suggests that under stress conditions, retinal neurons are able to use pyruvate for oxidative phosphorylation, but preferentially use lipids under normal conditions.

*Vldlr* ^*-/-*^ mice are deficient in both glucose and lipid uptake [7], while *Pparα* ^*-/-*^ mice are deficient in FAO but not glucose metabolism. *Vldlr* ^*-/-*^ and *Pparα* ^*-/-*^ mice had an equivalent decrease in NADH-linked respiration (Fig. 5e, f), suggesting similar rates of oxidative phosphorylation, which support the notion that lipids are an important substrate for oxidative phosphorylation in the retina. However, *Vldlr* ^*-/-*^ mice develop both neurodegeneration and pathological neovascularization [7, 15], while *Pparα* ^*-/-*^ mice undergo neurodegeneration similar to *Vldlr* ^*-/-*^ mice (Fig. 1c, e), but do not develop neovascular lesions [22]. These findings potentially suggest that there may be different roles for glucose and lipid metabolism in neuronal survival and neovascularization, or that neovascularization results from the extreme stress of both lipid and glucose deficiency in retinal neurons. The relative role of glucose versus lipid metabolism in the retina and in retinal disease states is an important topic of future investigation.

Paradoxically, although *Pparα* ^*-/-*^ mice do not develop neovascularization or vascular hyperpermeability characteristic of neovascular eye disease, PPARα activation alleviates neovascularization in *Vldlr* ^*-/-*^ mice and in ischemic proliferative retinopathy [7, 17, 22]. Further, *Pparα* ^*-/-*^ mice have increased retinal microvascular dysfunction in diabetic conditions [22]. This suggests that although PPARα does not suppress neovascularization in normal conditions, it suppresses it in pathological conditions, perhaps by improving retinal energy efficiency. We have also previously demonstrated that PPARα suppresses pathological Wnt signaling in diabetic and ischemic retinopathies [22], but found that expression of Wnt target genes was unchanged in *Pparα* ^*-/-*^ retinas (Additional file 9: Figure S9). This further suggests context-dependent roles for PPARα.

Metabolic dysfunction and resultant bioenergetic aberrations are now recognized as a factor in many aging-related neurodegenerative conditions. AMD is associated with mitochondrial dysfunction and energy deficiency [3, 6]. Impaired cholesterol metabolism may contribute to formation of senile plaques and neurofibrillary tangles in Alzheimer’s disease [4], and metabolic dysregulation has been linked to Parkinson’s disease [5]. Mitochondrial dysfunction in neurodegenerative disease may result in impaired ATP production, and may also increase mitochondrial generation of reactive oxygen species or initiate mitochondrial apoptosis [23]. We demonstrated here that PPARα activation improves mitochondrial efficiency and utilization of lipids as an energy source, which may therefore alleviate aging-related mitochondrial dysfunction. PPARα agonists are in common clinical use and are well-tolerated, and may therefore be an effective therapeutic approach for age-related neurodegeneration occurring secondary to metabolic dysregulation.

## Conclusions

Here we revealed a new role for endogenous PPARα in retinal neuronal energy metabolism, and demonstrated that utilization of lipids as a fuel source is critical to the survival of retinal neurons. These findings have three major implications: (i)PPARα is essential for retinal neuronal survival and lipid metabolism in physiological conditions; (ii) PPARα activation increases retinal energy efficiency and utilization of lipids as an energy substrate (iii) PPARα may be a new therapeutic target for AMD and other neurodegenerative conditions of metabolic origin. This work advances basic understanding of retinal energy metabolism and has significant translational potential.

## Methods

### Experimental animals

*Mice:* C57Bl/6 J, *Pparα* ^*-/-*^, and *Vldlr* ^*-/-*^ mice were originally purchased from Jackson Laboratories (Bar Harbor, ME, USA). Both *Pparα* ^*-/-*^ and *Vldlr* ^*-/-*^ mice were global deletions. Experimental animals were bred in-house. For all experiments, animals were maintained on a 12-hour light-dark cycle on a standard laboratory rodent diet (Purina 5053). Four-week-old WT and *Pparα* ^*-/-*^ mice were injected with either GFP- or PPARα-expressing adenovirus (1 μL of 2.52 × 10^9^ vector particles/mL) 4 weeks prior to sacrifice. *Vldlr* ^*-/-*^ mice and WT controls were injected intraperitoneally with 20 mg/kg fenofibric acid (AK Scientific, Union City, CA, USA) daily for 2 weeks prior to sacrifice. For terminal studies, animals were euthanized with carbon dioxide asphyxiation, 200 mg/kg pentobarbital (Virbac, Ft. Worth, TX, USA) or 300/60 mg/kg ketamine/xylazine (Virbac) as specified for each assay.

*Rats:* Sprague–Dawley rats were purchased from Charles River Laboratories (Wilmington, MA, USA) at 8 weeks of age and maintained on a 12-hour light-dark cycle. Rats were fed with control or fenofibrate-containing chow (Purina 5053) for 1 month prior to the experimental endpoint. Fenofibrate was purchased from Sigma-Aldrich (St. Louis, MO, USA) and blended homogenously with chow for a final dose of 20 mg/kg fenofibrate per day (Test Diet, Ft. Worth, TX, USA). Rats were euthanized with 200 mg/kg pentobarbital.

### Electroretinograms (ERGs)

ERG recordings were conducted in WT and *Pparα* ^*-/-*^ mice at 7, 9, 11, 26, and 52 weeks of age as described previously [15]. Briefly, mice were dark-adapted at least 12 hours prior to the procedure, and were then anesthetized with an 85/12 mg/mL ketamine/xylazine mixture. Pupils were dilated with Atropine (Alcon Laboratories, Ft. Worth, TX, USA) just prior to the procedure. ERG responses were recorded with a platinum needle electrode placed on the corneal surface, a reference electrode placed at the nasal formix, and a ground electrode on the foot. Fourteen responses to a light stimulus of 10 mSec with flash intervals of 20 sec were recorded. For quantitative analyses, the B-wave amplitude was measured between A- and B-wave peaks. N is expressed as number of animals per genotype, per time point.

### Optical coherence tomography (OCT)

OCT was used to measure total retinal thickness in WT and *Pparα* ^*-/-*^ mice at 13, 28, and 60 weeks of age. Mice were anesthetized and pupils dilated as described above. OCT images were taken using an Envisu R2000 imaging system (Bioptegen, Durham, NC, USA). Retinal scans were collected from both eyes of each mouse; 3–4 en face scans were collected from each retina, with each en face scan consisting of 100 B-scans and 1000 A-scans per B-scan. Total retinal thickness was measured using the manual digital caliper feature in the Envisu software, and was measured as the distance from the inner retinal nerve fiber layer to the outer retinal pigment epithelial layer. Measurements were made at 0°, 90°, 180°, and 270° on each retina at a radius of 500 μm. The 0° measurement on “OD” images and the 180° measurement on “OS” images were used for the temporal quadrant, and the opposite measurements were used for the nasal quadrant. N is expressed as number of animals per genotype, per time point.

### Cell death ELISA

A DNA fragmentation ELISA (Cell Death Detection ELISA; Roche Applied Science, Indianapolis, IN, USA) was used to quantify total retinal apoptosis in WT and *Pparα* ^*-/-*^ mice at 4, 8, 18 and 40 weeks of age and in 11-week-old WT and *Vldlr* ^*-/-*^ mice ± fenofibric acid as described previously [16, 24]. Mice were euthanized with carbon dioxide asphyxiation and retinas quickly excised. Retinas were homogenized in lysis buffer, incubated 30 minutes at room temperature, and spun down to generate a cytoplasmic fraction. The cytoplasmic fraction was then subjected to ELISA analysis as per the manufacturer’s protocol, and relative fragmentation was expressed as OD (405/490 nm) normalized by total protein content as measured by RC/DC assay (Biorad, Hercules, CA, USA) OD (750 nm). N is expressed as number of retinas per genotype, per time point.

### Western blotting

Western blotting was used to measure retinal GFAP levels in WT and *Pparα* ^*-/-*^ mice at 8, 15, and 40 weeks of age as described previously [25]. Mice were euthanized with carbon dioxide asphyxiation and retinas were snap-frozen in liquid nitrogen. One retina per animal was homogenized in Holt’s lysis buffer, and 10 μg of protein were resolved by SDS-PAGE and transferred to a nitrocellulose membrane. Membranes were probed with GFAP antibody (Sigma; Cat # G3893; RRID AB_477010) and subsequently reprobed with HRP-conjugated β-actin (Santa Cruz Biotechnology, Santa Cruz, CA, USA; Cat # sc-47778 HRP; RRID AB_2714189). Band density was quantified using Alpha Imager software (San Jose, CA, USA). N is expressed as number of retinas per genotype, per time point.

### Isolectin staining, flatmount, and vascular density quantification

To image retinal vasculature in WT and *Pparα* ^*-/-*^ mice, isolectin staining was used as described previously [26]. Mice were euthanized with carbon dioxide asphyxiation and whole eyes were fixed in 4% paraformaldehyde for 1 hour and washed with PBS for 10 minutes, three times. Retinas were then dissected and penetrated with 70% ice-cold ethanol at room temperature for 30 minutes, and subsequently penetrated in 1% TPBS (Triton X-100) for 1 hour at room temperature. Retinas were then stained with fluorescent *Griffonia simplicifolia* isolectin B4 (Invitrogen, Carlsbad, CA, USA) overnight. The stained retinas were washed in three times in PBS for 30 minutes, and subsequently flat-mounted in glycerol media for fluorescent imaging.

To quantify vascular density, single fields of the superficial and deep vascular plexuses in the central and peripheral retina (four fields per retina, one field per quadrant) were imaged at 200× magnification (dimensions: 1388 × 1040). Prior to analysis, the background of each image was selected and cropped manually in Image J using the freehand tool. The background color was set as black. The image was converted to 8 bit before using “Analyze HUVEC Fluo” in the Angiogenesis Analyzer tool for ImageJ. (http://image.bio.methods.free.fr/ImageJ/?Angiogenesis-Analyzer-for-ImageJ). Total vessel length per field, number of junctions per field, and number of meshes per field were used as parameters for the evaluation of retinal vascular network. N is expressed as the number of retinas quantified per genotype, per time point.

### Fatty acid oxidative capacity

To measure fatty acid oxidative capacity of WT and *Pparα* ^*-/-*^ retinas, oxygen consumption rates after addition of fatty acid BSA-conjugated palmitate or BSA vehicle were measured using a Seahorse XFe96 Flux Analyzer® as described previously [7], with minor modifications. Mice were euthanized with ketamine/xylazine, whole retinas were isolated and 1 mm punch biopsies were loaded into a 96-well spheroid plate. Retinal punches were incubated in assay media consisting of DMEM 5030 supplemented with 20 mM glucose, 5 mM pyruvate, 2 mM glutamine, 10 mM HEPES, and 0.5 mM carnitine (Sigma), and 30 μL BSA-conjugated palmitate or BSA vehicle control (Seahorse/Agilent, Santa Clara, CA) were added to a final concentration of 168 μM just prior to commencing the assay. The Seahorse XF Mito Stress Test Kit® was used to assess mitochondrial respiration as described in the manufacturer’s protocol with minor modifications. Oligomycin was injected in Port A to a final concentration of 3 μM, trifluoromethoxyphenylhydrazone (FCCP) was injected in Ports B and C to a final concentration of 1.5 μM, and rotenone/antimycin A (RAA) was injected in Port D to a final concentration of 4 μM rotenone/4 μM antimycin A. Basal respiration was calculated by subtracting non-mitochondrial respiration (rate after injection of RAA) from the basal rate. Maximal respiration was calculated by subtracting non-mitochondrial respiration from the rate after injection of FCCP. Fold change in basal and maximal respiratory rates was calculated by dividing basal and maximal rates of palmitate-treated punches by that of BSA-treated punches. To determine if increased OCR in palmitate-treated retinas was due to fatty acid oxidation, control punches were treated with 40 μM Cpt1a inhibitor etomoxir (Sigma) or vehicle control for 1 hour prior to commencing the assay. N is expressed as the number of retinal punches per treatment group.

### Pyruvate oxidative capacity

To determine whether pyruvate was used as an oxidizable substrate in WT and *Pparα* ^*-/-*^ retinas, the retinal OCR was measured after addition of pyruvate using a Seahorse XFe96 Flux Analyzer®. Mice were euthanized with ketamine/xylazine, whole retinas were isolated and 1 mm punch biopsies were loaded into a 96-well spheroid plate. Retinal punches were incubated in assay media consisting of DMEM 5030 supplemented with 5 mM glucose, 0.5 mM pyruvate, 2 mM glutamine, and 10 mM HEPES. Assay media (vehicle) control or high-pyruvate-containing assay media were injected into port A to a final concentration of 5 mM pyruvate in pyruvate-injected punches, and 0.5 mM pyruvate in vehicle-injected punches. Oligomycin was injected into port B to a final concentration of 1 μM, FCCP was injected into port C to a final concentration of 2 μM, and RAA was injected in Port D to a final concentration of 2 μM rotenone/2 μM antimycin A. Maximal respiration was calculated by subtracting non-mitochondrial respiration from the rate after injection of FCCP. Fold change in maximal respiratory rate was calculated by dividing the maximal rate of pyruvate-injected punches by that of vehicle-injected punches for both genotypes. N is expressed as the number of retinal punches per treatment group.

### Isolation of retinal mitochondria

Retinal mitochondria were isolated as described previously [27] with minor modifications from rats fed fenofibrate (20 mg/kg) or vehicle 1 month prior to sacrifice. Rats were euthanized with pentobarbital. Retinas were rapidly excised and homogenized in 5-mL ice-cold buffer A (210 mM mannitol, 70 mM sucrose, 1.0 mM EDTA, 5.0 mM MOPS, pH 7.4) with four retinas per preparation. Retinas were then subjected to five passes with a motor-driven Potter–Elvehjem tissue grinder and spun at 500 *g* for 5 min at 4 °C. The supernatant was subsequently collected and passed through a cheesecloth, then spun again at 500 *g* for 10 min. The resultant mitochondrial pellet was resuspended in 40 μL of buffer A and protein concentration determined by BCA assay (Pierce, Green Island, NY, USA) according to the manufacturer’s protocol.

### Mitochondrial respiration in isolated mitochondria

Mitochondrial respiration was measured in retinal mitochondria from rats fed fenofibrate or vehicle as described previously [27]. Mitochondria were diluted to 0.25 mg/mL in buffer B (210 mM mannitol, 70 mM sucrose, 5.0 mM KH_2_PO_4_, and 10 mM MOPS, pH 7.4) containing either 0.1 mM pyruvate/1.0 mM malate or 30 μM palmitoylcarnitine/0.1 mM malate/0.5 mg/mL BSA. Respiration was measured at 20 °C using a fiber optic oxygen measurement system that employs the fluorescence lifetime technique (Instech, Plymouth Meeting, PA, USA). State 3 respiration was initiated by addition of 0.5 mM ADP (Sigma). N is expressed as the number of mitochondrial preparations per treatment group, with four retinas per preparation.

### Mitochondrial respiration in intact retinas

To determine whether mitochondrial respiration was changed in WT and *Pparα* ^*-/-*^ retinas after onset of neurodegeneration, retinal oxygen consumption rate (OCR) was measured in 8- and 12-week-old retinas using a Seahorse XFe96 Flux Analyzer®. Mice were euthanized with ketamine/xylazine, whole retinas were isolated and 1 mm punch biopsies were loaded into a 96-well spheroid plate. Retinal punches were incubated in assay media consisting of DMEM 5030 supplemented with 12 mM glucose, 5 mM pyruvate, 2 mM glutamine and 10 mM HEPES. Oligomycin was injected into port A to a final concentration of 2 μM, FCCP was injected into port B and C to a final concentration of 1 μM, and Rotenone/Antimycin A (RAA) was injected in Port D to a final concentration of 2 μM rotenone/2 μM Antimycin A. Basal respiration was calculated by subtracting non-mitochondrial respiration from the basal rate prior to injection of Oligomycin. Maximal respiration was calculated by subtracting non-mitochondrial respiration from the rate after injection of FCCP. N is expressed as the number of retinal punches per treatment group.

### Adenovirus preparation

PPARα-expressing adenovirus was generated previously [17]. Ad-GFP prepared previously was used as a control. Preparation, amplification, and titration of the recombinant adenoviruses was performed as described previously [28, 29].

### *In vitro* PPARα knockdown and extracellular flux analysis

R28 rod precursor cells were a kind gift of Dr. Gail Seigel [30]. Cells were cultured in Dulbecco’s modified Eagle’s medium (DMEM) containing 1 mM D-glucose, 10% heat-inactivated fetal bovine serum (FBS; Cellgro, Manassas, VA, USA) and 1% antibiotic-antimycotic solution (Cellgro).

#### Transfection

Smartpool siRNA (Dharmacon, Lafayette, CO, USA) targeting rat PPARα or scrambled control was delivered into R28 cells by Hiperfect (Qiagen) by reverse transfection following the manufacturer’s protocol. Briefly, 5000 or 120000 R28 cells/well were seeded in an Agilent Seahorse XF96 cell culture microplate or a 12-well plate, respectively. At the same time, cells received 50nM siRNA diluent mixed with Hiperfect. Forty-eight hours after transfection, knockdown efficiency was detected by real-time PCR and oxygen consumption rate was measured using a Seahorse XFe96 Flux Analyzer®.

#### Seahorse extracellular flux analysis

Forty-eight hours after transfection, oxygen consumption rates were measured using a Seahorse XFe96 Flux Analyzer® following the manufacturer’s protocol. Oligomycin was injected into port A to a final concentration of 2 μM, FCCP was injected into port B to a final concentration of 6 μM, and Rotenone/Antimycin A (RAA) was injected in Port C to a final concentration of 4 μM rotenone/4 μM Antimycin A. Maximal respiration was calculated by subtracting non-mitochondrial respiration from the rate after injection of FCCP. After measurement of oxygen consumption rates, cells were lysed in 30 μl 1X SDS loading buffer. The lysate was resolved by SDS-PAGE and transferred to a nitrocellulose membrane. Membranes were probed with HRP-conjugated anti-β-Actin (Santa Cruz Biotechnology). Band density was quantified using Image J. OCR data was normalized by the density of β-Actin. N is expressed as the number of wells per treatment group.

### NADH oxidation assay

To quantify mitochondrial NADH-linked respiration, the consumption of exogenously added NADH was measured spectrophotometrically in WT vs. *Pparα* ^*-/-*^ retinas and in WT and *Vldlr* ^*-/-*^ retinas ± fenofibric acid as described previously [31] with minor modifications. Animals were euthanized with pentobarbital, and retinas rapidly excised and snap-frozen in liquid nitrogen. Retinas were homogenized in 250 μL 25 mM MOPS/1 mM EDTA pH 7.4 and sonicated. Prior to the reaction, retinal homogenates were diluted 1:20 in assay buffer (25 mM MOPS/10 mM KCl, pH 7.4). Exogenous NADH (100 μM; Sigma) was added and its oxidation rate monitored spectrophotometrically at 340 nm. Protein concentration of retinal homogenates was measured using a BCA assay as described above. Data are expressed as ratio of nmol NADH consumed per minute per mg of total retinal protein in control versus experimental group. To verify that observed changes in NADH consumption were due to NADH-linked respiration, complex I inhibitor rotenone (3.5 μM; Sigma) was added to the reaction described above. All assays were performed at room temperature. N is expressed as the number of retinas per genotype, per time point.

### Reverse transcription and quantitative real-time PCR

Lipid transporter and FAO enzyme RNA levels were measured in WT vs. *Pparα* ^*-/-*^ retinas using quantitative real-time PCR (qRT-PCR) as described previously [7], with minor modifications. Briefly, mice were euthanized with carbon dioxide asphyxiation and retinal RNA was isolated using a Qiazol (Qiagen, Valencia, CA, USA) extraction, purified using a PureLink® RNA Mini Kit (Qiagen) and treated with DNase I (Qiagen) to remove contaminating genomic DNA. DNase-treated RNA was then converted into cDNA using iScript® reverse transcriptase (BioRad, Hercules, CA, USA). PCR primers for target genes and housekeeping gene *β-actin* were designed using NCBI Primer Blast and OligoCalc software. Primer sequences are shown in Table 1. Quantitative analysis of gene expression was conducted using a BioRad CFX96® system with a SYBR Green Master Mix kit (BioTools, Jupiter, FL, USA) and target gene expression was calculated relative to *β-actin* using the ∆cT method. For in vivo studies, N is expressed as the number of retinas per genotype. For in vitro studies, N is expressed as the number of wells per group.

**Table 1:** Primer sequences

Gene	Forward 5′ – 3′	Reverse 5′ – 3′
*β-Actin*	CGGTTCCGATGCCCTGAGGCTCTT	CGTCACACTTCATGATGGAATTGA
*Cpt1a*	TGGCATCATCACTGGTGTGTT	GTCTAGGGTCCGATTGATCTTTG
*Fabp3*	GTGACAGCAGATGACCGGAA	TGCCATGAGTGAGAGTCAGGA
*Slc25a20*	CTATGTTCGCCGTGTGCTTC	GAAGCCTGAATCTGCAGTAAGC
*Acaa2*	GAACACCCTTTGGAGCTTACG	CACATGCCTCGCCAGGTATG
*Acadl*	GCTTGGCATCAACATCGCAG	ATTCGCAATATAGGGCATGACAA
*Acadm*	AGTACCCGTTCCCTCTCATCA	TACACCCATACGCCAACTCTT
*Acads*	ACAGTGGATCACCCCTTTCAC	ACTGAGGGCAAAACAGCCG
*Acadvl*	GGAGGACGACACTTTGCAGG	AGCGAGCATACTGGGTATTAGA
*Acot13*	TAGAGTTTTGGAAAAGGTGACGC	GGTTGCTGTCAAGCCTCCAT
*Acox1*	TAACTTCCTCACTCGAAGCCA	AGTTCCATGACCCATCTCTGTC
*Acsl1*	CCATCTTCCCTGTGGTTCCC	ACCCAGGCTCGACTGTATCT
*Hadha*	GGCCTTGTGGAAAAGCTGAC	GATAACCAGCGTCACTGCCT
*Hmgcs2*	GAAGAGAGCGATGCAGGAAAC	GTCCACATATTGGGCTGGAAA
*Hsd17b4*	GTTGCCGGTTTTGAGAAGCC	TCCTGGATACACTGGTTTGGC
*Gsta3*	AGGAACAAACCAGGAACCGT	GCTTCTCAGCGCTTTCAGGA
*Msra*	GTAACAGCCAAACACCATGTCA	GACCCAGAACTTGCGCTCAG
*Prdx5*	CCGATCAAGGTGGGAGATGC	CTTGGCCTTCAGAGCTCCAG
*Prdx6*	TTTCCTGGGAGATTCCTGCTGAG	TGCCACTTCATCTGCTTATTTAGTT
*Pparα*	GGTGCATTTGGGCGTAACTC	AGTCTTGGCTCGCCTCTAAG

### Transmission electron microscopy (TEM)

TEM was utilized to compare mitochondrial morphology in WT versus *Pparα* ^*-/-*^ retinas as described previously [32]. Mice were euthanized with carbon dioxide asphyxiation and perfused with 4% paraformaldehyde for 30 minutes. Eyes were removed and fixed overnight, and retinas subsequently excised. Tissue sections were generated using a microtome (Reichert-Jung Ultracut E Microtome, American Instrument, Haverhill, MA, USA) with a diamond knife. Sections (600–800 Å) were collected on copper 75/300 mesh grids for conventional TEM analysis and stained with 2% uranyl acetate and Reynolds’ lead citrate to examine the morphology of photoreceptor mitochondria. Sections were viewed with a transmission electron microscope (JEOL 100CX; JEOL USA Inc., Peabody, MA, USA) at an acceleration voltage of 50 keV. Digital images were collected and stored on a computer for subsequent viewing and analysis. Three retinas from three separate animals were used for each genotype, and over 100 retinal mitochondria were imaged for each animal.

### Mass spectrometry

Mass spectrometry was utilized for targeted quantitative proteomic analysis of metabolic pathways in WT versus *Pparα* ^*-/-*^ retinas as previously performed albeit with minor modifications [19].

#### Sample preparation

Mice were euthanized with carbon dioxide asphyxiation and PBS-perfused. Retinas were dissected and snap-frozen in liquid nitrogen. Both retinas from each animal were homogenized in 200 μL Holt’s lysis buffer and sonicated, then spun at 500 *g* for 5 min at 4 °C, and the supernatant was collected. Protein was quantified using a BioRad detergent compatible kit (Hercules, CA) according to the manufacturer’s protocol. The volume equivalent to 60 μg protein was taken for analysis, and 8 pmol of bovine serum albumin were added as a non-endogenous internal standard. Samples were mixed, heated and cooled to room temperature. Acetone (1 mL) was added and proteins were precipitated overnight at –20 °C. The resultant pellet was reconstituted in 60 μL of sample loading buffer and a 20 μL aliquot was resolved by short-run gel electrophoresis (1.5 cm into gel), gel-fixed, and stained. Each lane was cut as a single sample, washed, reduced with DTT, alkylated with idoacetamide, and digested overnight with trypsin. The peptides from the digestion were extracted, evaporated to dryness, and reconstituted in 150 μL 1% acetic acid for analysis.

#### LC-tandem mass spectrometry analysis

An Eskigent nanoflow system (Sciex, Framingham, MA, USA) was used for liquid chromatography (LC) and ThermoScientific TSQ Vantage triple quadrupole was used for mass spectrometry (Thermo Scientific, San Jose, CA, USA). A 75 μM × 11 cm column packed with Phenometrix Jupiter C18 was used. Sample aliquots (6 μL) were injected for each analysis. Peptides were eluted with a linear gradient of acetonitrile in water with 0.1% formic acid. For data analysis, the open-source program Skyline (developed by Dr. Michael MacCoss, University of Washington) was used. Total protein responses were calculated as the geometric mean of the abundance of peptides measured for each protein, and normalized to bovine serum albumin (BSA) total protein response. The final abundance of each protein was calculated as pmol/100 μg total protein based on the ratio in relation to BSA and the amount of BSA added, and data were expressed as the amount of protein in experimental groups relative to control.

### Statistics

Results are expressed as mean ± SEM. A two-tailed, unpaired Student’s *t* test was used for two-group comparisons, and a one-way ANOVA with Tukey’s posthoc comparison was used for studies with more than two experimental groups. *P* values of less than 0.05 were considered statistically significant.

## Additional files

Additional file 1: Figure S1. (A) Multiple intensity electroretinograms confirmed that retinal function was suppressed in 36-week-old *Pparα* ^*-/-*^ mice relative to wild-type (WT) when multiple flicker durations were utilized (n = 5 mice/genotype). (B) Representative scotopic (rod) electroretinogram traces from a 36-week-old WT mouse at multiple flicker durations. (C) Representative scotopic (rod) electroretinogram traces from a 36-week-old *Pparα* ^*-/-*^ mouse at multiple flicker durations. (D) Representative photopic electroretinogram trace from a 36-week-old WT mouse. (E) Representative photopic electroretinogram trace from a 36-week-old *Pparα* ^*-/-*^ mouse. (F) Retinal cell death ELISA demonstrated that intravitreal injection of PPARα-expressing adenovirus alleviated retinal apoptosis in 8-week-old *Pparα* ^*-/-*^ mice (WT ad-GFP n = 5; *Pparα* ^*-/-*^ ad-GFP n = 6; *Pparα* ^*-/-*^ ad-PPARα n = 7). Data are expressed as mean ± SEM. *P ≤ 0.05; ***P ≤ 0.001; ****P ≤ 0.0001, unpaired Student’s *t* test (A) or one-way ANOVA with Tukey’s post-hoc comparison (F). Figure S1 pertains to Fig. 1 of the main text. (TIF 21187 kb)

Additional file 2: Figure S2. (A–C) Full gels for immunoblots shown in Fig. 1d. Gels are loaded alternating wild-type (WT) and *Pparα* ^*-/-*^ retinal lysates of specified ages. Rectangles denote representative bands included in figure 1D. (D) At 15 and 40 weeks of age, a 45 kDa isoform of GFAP was present, and was increased in *Pparα* ^*-/-*^ retinas relative to age-matched WT controls (15 weeks n = 5 retinas/genotype; 40 weeks n = 6 retinas/genotype). **P ≤ 0.01, unpaired two-tailed Student’s *t* test. Figure S2 pertains to Fig. 1 of the main text. (TIF 24547 kb)

Additional file 3: Figure S3. (A) Representative images of isolectin-labeled microvessels in retinal wholemount and single fields of superficial and deep vascular plexuses in central and peripheral retina of 8-week-old wild-type (WT) retinas. (B) Representative images of 8-week-old *Pparα* ^*-/-*^ retinas. (C) Modestly decreased total vessel length in the central retinal region of the deep vascular plexus in *Pparα* ^*-/-*^ retinas. (D) Modestly decreased number of vascular junctions in the central retinal region of the deep vascular plexus in *Pparα* ^*-/-*^ retinas. (E) Decreased number of meshes in central region of deep vascular plexus of *Pparα* ^*-/-*^ retinas (adult retinas do not have meshing in the superficial plexus) (n = 10 retinas/genotype). *P ≤ 0.05; **P ≤ 0.01, unpaired two-tailed Student’s *t* test. Figure S3 pertains to Fig. 2 of the main text. (TIF 31492 kb)

Additional file 4: Figure S4. (A, B) Palmitate (Palm)-induced increases in oxygen consumption rate (OCR) in wild-type (WT) retinas were reversed by Cpt1a inhibitor etomoxir (Eto), suggesting that increased OCR with palm is due to increased fatty acid oxidation (BSA n = 12; Palm n = 10; Palm + Eto n = 12). (C, D) Palm and Eto do not change OCR in *Pparα* ^*-/-*^ retinas (BSA n = 9; Palm n = 12; Palm + Eto n = 9). (E) RNA levels of fatty acid oxidation enzymes are unchanged in 4-week-old *Pparα* ^*-/-*^ retinas relative to age-matched WT (n = 5). Please refer to Additional file 10: Table S1 for full names of genes presented in Additional file 3: Figure S3C. *P ≤ 0.05, unpaired two-tailed Student’s *t* test. Additional file 3: Figure S3 pertains to Fig. 3 of the main text. (TIF 12372 kb)

Additional file 5: Figure S5. Representative oxygen consumption trace for pyruvate substrate use experiment in isolated retinal mitochondria from fenofibrate-treated rats shown in Fig. 4e of main text, demonstrating no change in oxygen consumption with fenofibrate treatment when pyruvate is provided as an oxidizable substrate (n = 4). Figure S5 pertains to Fig. 4 of the main text. (TIF 6362 kb)

Additional file 6: Figure S6. (A-B) Seahorse extracellular flux analysis demonstrated that intravitreal injection of PPARα-expressing adenovirus restored deficient retinal mitochondrial respiration in 8-week-old *Pparα* ^*-/-*^ mice (wild-type (WT) ad-GFP n = 19; *Pparα* ^*-/-*^ ad-GFP n = 25; *Pparα* ^*-/-*^ ad-PPARα n = 19). (C) Seahorse extracellular flux analysis demonstrated that siRNA knockdown of PPARα in R28 rod precursor cells decreased mitochondrial respiration (siScr n = 7; siPPARα n = 6). (D) siRNA knockdown of PPARα reduces mRNA levels by ~50% in R28 cells (n = 4). (E) NADH oxidation assay revealed that NADH oxidation was decreased in 8-week-old *Pparα* ^*-/-*^ retinas relative to age-matched WT, which was significantly abrogated by complex I inhibitor rotenone. NADH oxidation was unchanged in *Pparα* ^*-/-*^ retinas relative to WT in rotenone-inhibited reactions (WT n = 10; *Pparα* ^*-/-*^ n = 9). B: *P ≤ 0.05 vs. WT-GFP, ^#^ *P* ≤ 0.05 vs. *Pparα* ^*-/-*^ + GFP, one-way ANOVA with Tukey’s post-hoc comparison. C–E: **P ≤ 0.01; ***P ≤ 0.001; ****P ≤ 0.0001, unpaired two-tailed Student’s *t* test. Figure S6 pertains to Fig. 5 of the main text. (TIF 12715 kb)

Additional file 7: Figure S7. (A) Heatmap representation and gene symbols of proteins measured by proteomic analysis. Please refer to Additional file 10: Table S1 for full gene names. (B) Fatty acid oxidation enzymes are unchanged in *Pparα* ^*-/-*^ retinas relative to wild-type with exception of Hadha, which is upregulated. (C) Glycolytic enzymes Hk1, Ldhb, Pfkm, Pgam2, and Tpi are upregulated in *Pparα* ^*-/-*^ retinas. (D) Krebs cycle enzymes are unchanged in *Pparα* ^*-/-*^ retinas. (E) Electron transport chain enzymes are unchanged in *Pparα* ^*-/-*^ retinas. (F) Antioxidant enzymes Gsta3, Gsm1, Msra, Prdx5, Prdx6, Sod1, and Txnrd1 are upregulated in *Pparα* ^*-/-*^ retinas. WT n = 6, *Pparα* ^*-/-*^ n = 4. *P ≤ 0.05; **P ≤ 0.01; ***P ≤ 0.001, two-tailed unpaired Student’s *t* test. Figure S7 pertains to Fig. 6 of the main text. (TIF 28635 kb)

Additional file 8: Figure S8. At 4 weeks of age, antioxidant enzymes upregulated at 40 weeks (S7F) are unchanged in *Pparα* ^*-/-*^ retinas relative to age-matched wild-type. For full gene names refer to Additional file 10: Table S1. n = 5 retinas/genotype. Statistically insignificant as indicated by *P* ≥ 0.05, two-tailed unpaired Student’s *t* test. Figure S8 pertains to Fig. 6 of the main text. (TIF 7076 kb)

Additional file 9: Figure S9. Retinal expression of Wnt target genes was unchanged in *Pparα* ^*-/-*^ retinas relative to wild-type at 8 weeks of age. N = 5 retinas/genotype. Statistically insignificant as indicated by *P* ≥ 0.05, two-tailed unpaired Student’s *t* test. (TIF 6448 kb)

Additional file 10: Table S1. Genes presented in Figures S4, S7 and S8. (DOCX 124 kb)
