# Supplementary figures and images for: PPARα is essential for retinal lipid metabolism and neuronal survival
Source: BMC Biol. 2017 Nov 28;15:113. doi: 10.1186/s12915-017-0451-x (PMC5706156; doi:10.1186/s12915-017-0451-x)

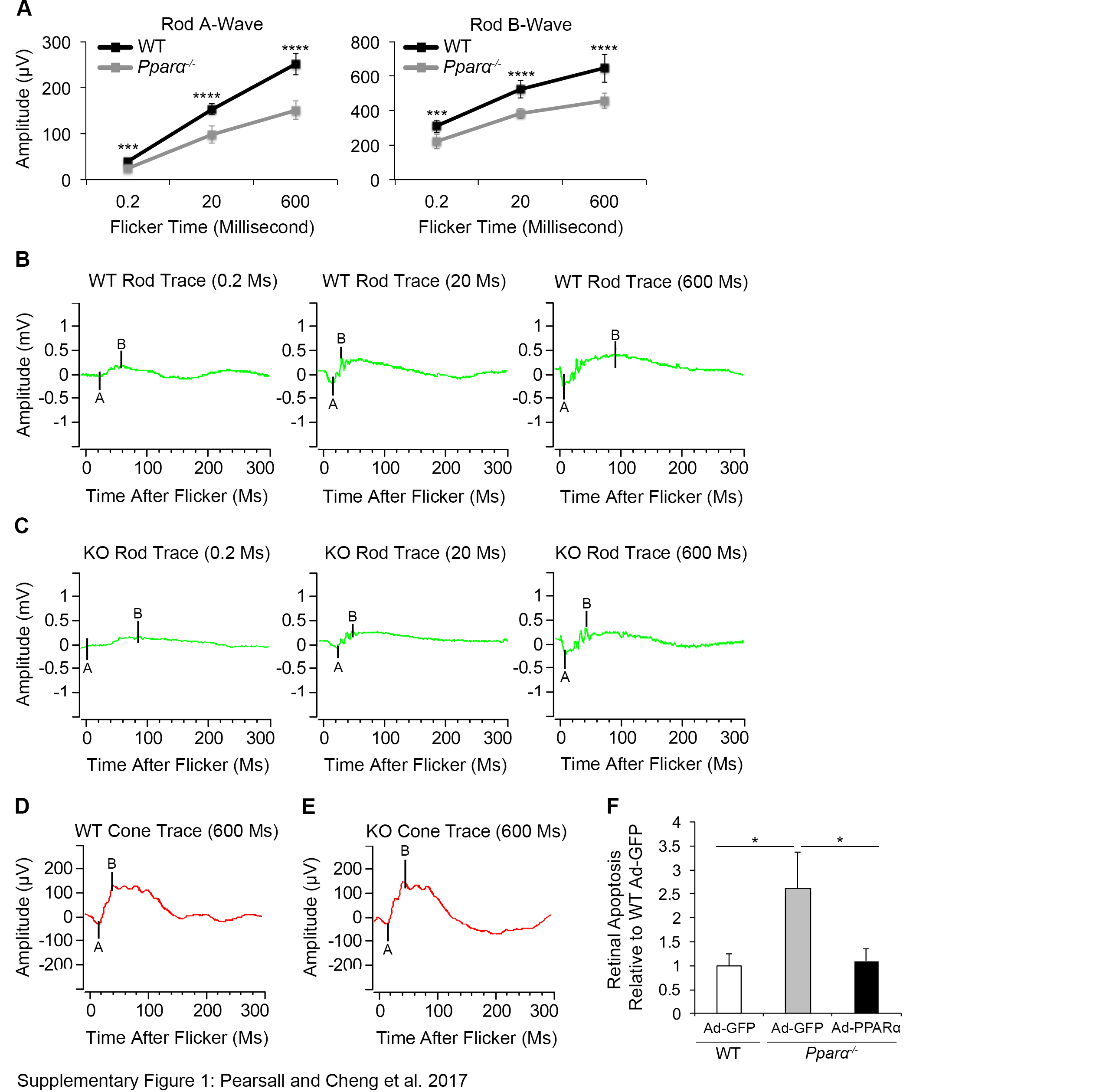

Supplement: Supplementary file 1 — (A) Multiple intensity electroretinograms confirmed that retinal function was suppressed in 36-week-old Pparα -/- mice relative to wild-type (WT) when multiple flicker durations were utilized (n = 5 mice/genotype). (B) Representative scotopic (rod) electroretinogram traces from a 36-week-old WT mouse at multiple flicker durations. (C) Representative scotopic (rod) electroretinogram traces from a 36-week-old Pparα -/- mouse at multiple flicker durations. (D) Representative photopic electroretinogram trace from a 36-week-old WT mouse. (E) Representative photopic electroretinogram trace from a 36-week-old Pparα -/- mouse. (F) Retinal cell death ELISA demonstrated that intravitreal injection of PPARα-expressing adenovirus alleviated retinal apoptosis in 8-week-old Pparα -/- mice (WT ad-GFP n = 5; Pparα -/- ad-GFP n = 6; Pparα -/- ad-PPARα n = 7). Data are expressed as mean ± SEM. *P ≤ 0.05; ***P ≤ 0.001; ****P ≤ 0.0001, unpaired Student’s t test (A) or one-way ANOVA with Tukey’s post-hoc comparison (F). Figure S1 pertains to Fig. 1 of the main text. (TIF 21187 kb) [file 12915_2017_451_MOESM1_ESM.tif]

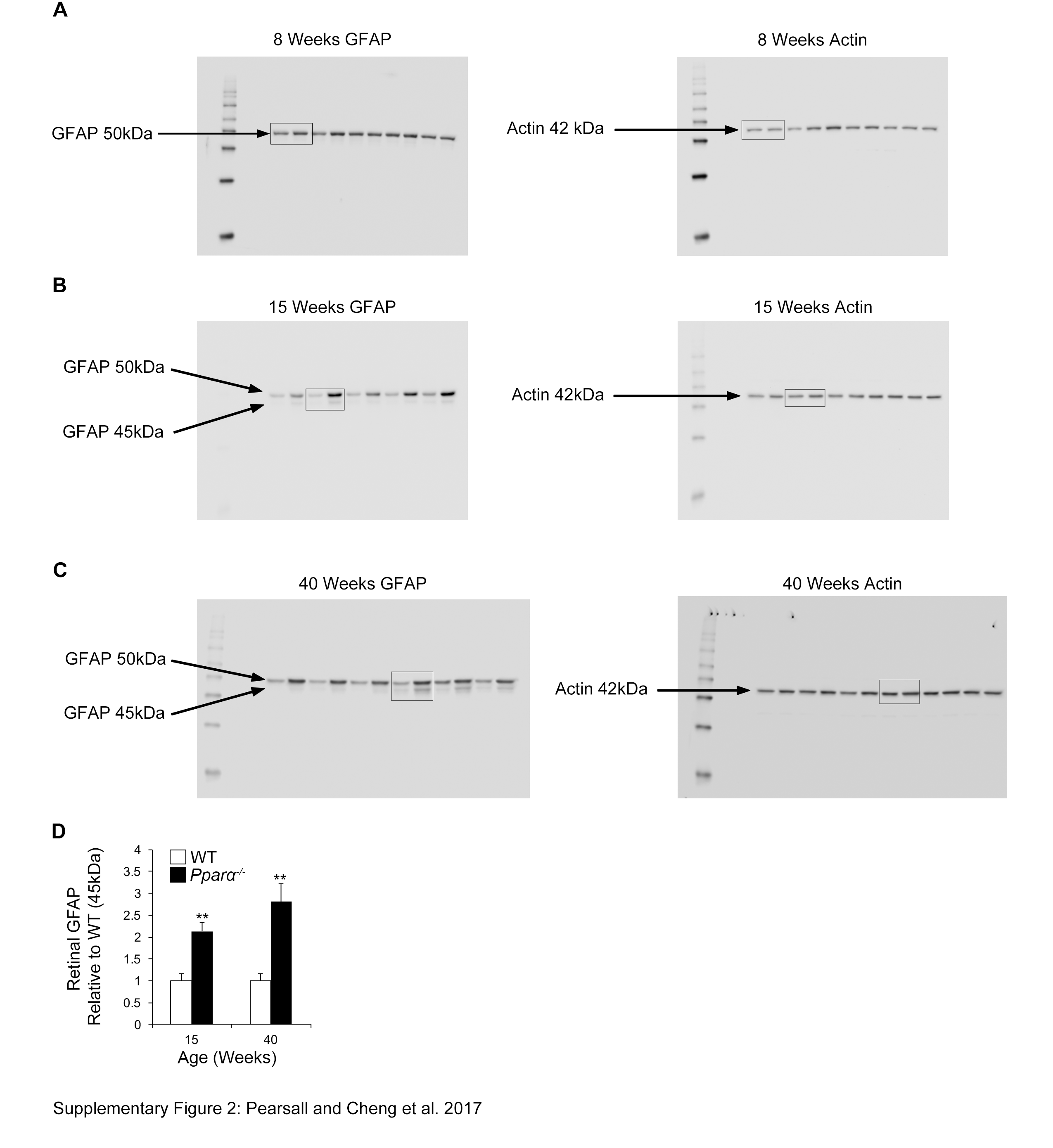

Supplement: Supplementary file 2 — (A–C) Full gels for immunoblots shown in Fig. 1d. Gels are loaded alternating wild-type (WT) and Pparα -/- retinal lysates of specified ages. Rectangles denote representative bands included in figure 1D. (D) At 15 and 40 weeks of age, a 45 kDa isoform of GFAP was present, and was increased in Pparα -/- retinas relative to age-matched WT controls (15 weeks n = 5 retinas/genotype; 40 weeks n = 6 retinas/genotype). **P ≤ 0.01, unpaired two-tailed Student’s t test. Figure S2 pertains to Fig. 1 of the main text. (TIF 24547 kb) [file 12915_2017_451_MOESM2_ESM.tif]

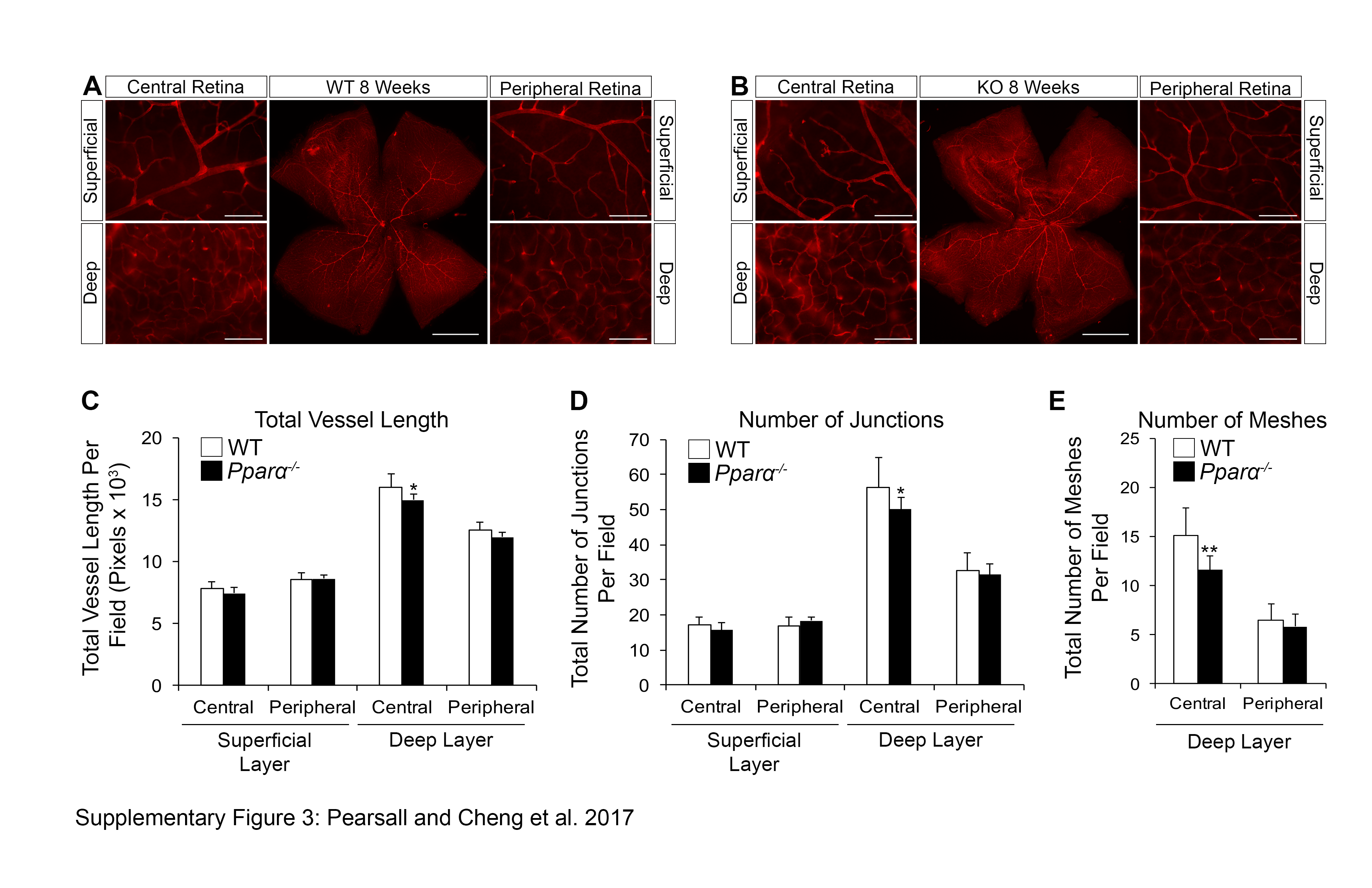

Supplement: Supplementary file 3 — (A) Representative images of isolectin-labeled microvessels in retinal wholemount and single fields of superficial and deep vascular plexuses in central and peripheral retina of 8-week-old wild-type (WT) retinas. (B) Representative images of 8-week-old Pparα -/- retinas. (C) Modestly decreased total vessel length in the central retinal region of the deep vascular plexus in Pparα -/- retinas. (D) Modestly decreased number of vascular junctions in the central retinal region of the deep vascular plexus in Pparα -/- retinas. (E) Decreased number of meshes in central region of deep vascular plexus of Pparα -/- retinas (adult retinas do not have meshing in the superficial plexus) (n = 10 retinas/genotype). *P ≤ 0.05; **P ≤ 0.01, unpaired two-tailed Student’s t test. Figure S3 pertains to Fig. 2 of the main text. (TIF 31492 kb) [file 12915_2017_451_MOESM3_ESM.tif]

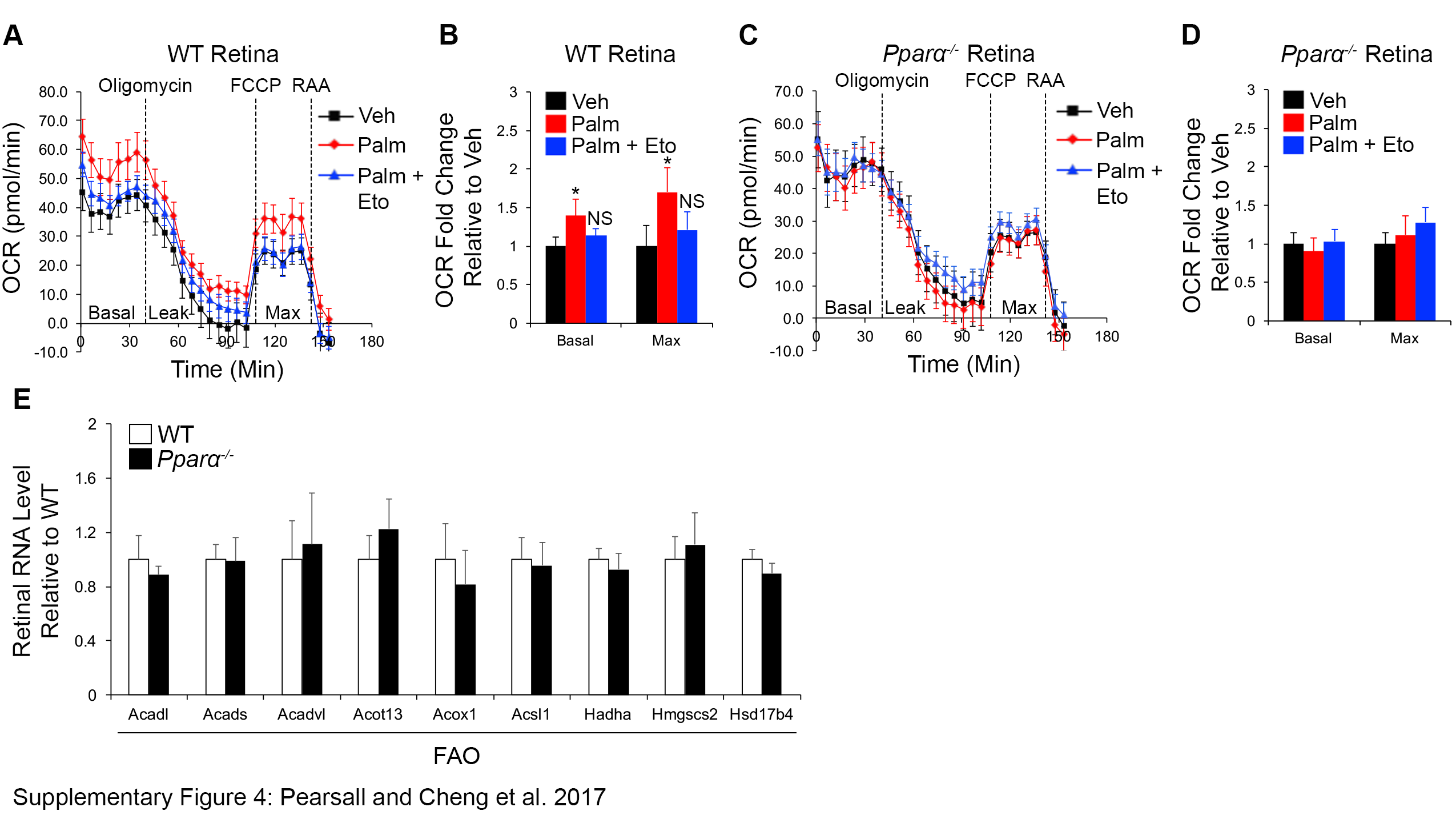

Supplement: Supplementary file 4 — (A, B) Palmitate (Palm)-induced increases in oxygen consumption rate (OCR) in wild-type (WT) retinas were reversed by Cpt1a inhibitor etomoxir (Eto), suggesting that increased OCR with palm is due to increased fatty acid oxidation (BSA n = 12; Palm n = 10; Palm + Eto n = 12). (C, D) Palm and Eto do not change OCR in Pparα -/- retinas (BSA n = 9; Palm n = 12; Palm + Eto n = 9). (E) RNA levels of fatty acid oxidation enzymes are unchanged in 4-week-old Pparα -/- retinas relative to age-matched WT (n = 5). Please refer to Additional file 10: Table S1 for full names of genes presented in Additional file 3: Figure S3C. *P ≤ 0.05, unpaired two-tailed Student’s t test. Additional file 3: Figure S3 pertains to Fig. 3 of the main text. (TIF 12372 kb) [file 12915_2017_451_MOESM4_ESM.tif]

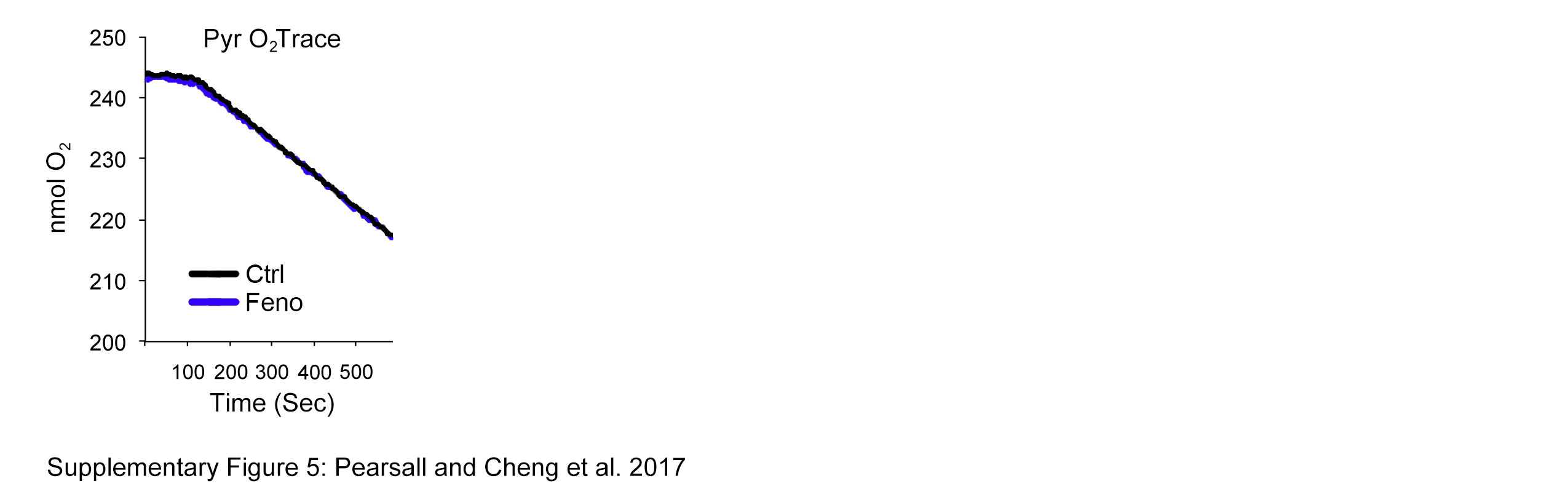

Supplement: Supplementary file 5 — Representative oxygen consumption trace for pyruvate substrate use experiment in isolated retinal mitochondria from fenofibrate-treated rats shown in Fig. 4e of main text, demonstrating no change in oxygen consumption with fenofibrate treatment when pyruvate is provided as an oxidizable substrate (n = 4). Figure S5 pertains to Fig. 4 of the main text. (TIF 6362 kb) [file 12915_2017_451_MOESM5_ESM.tif]

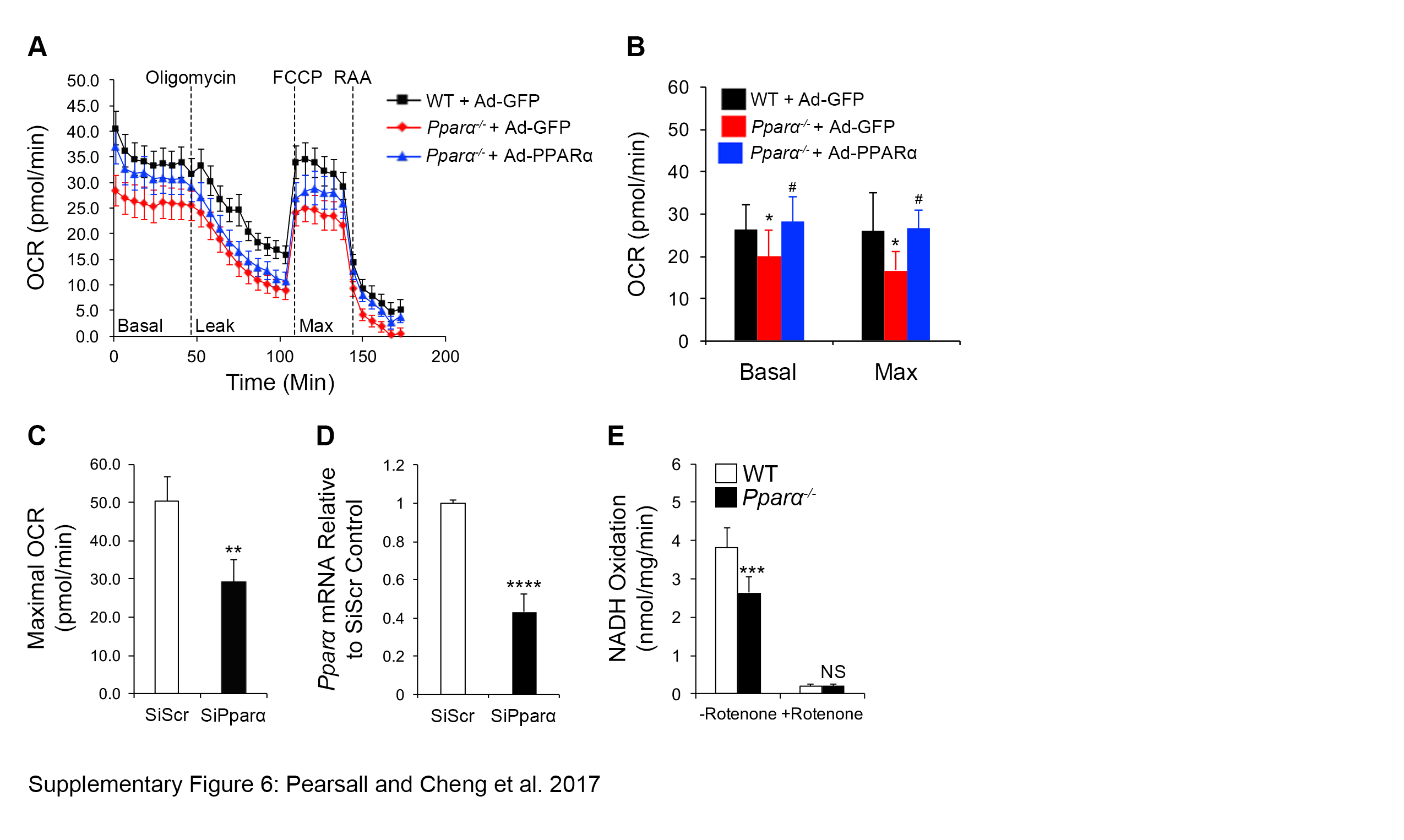

Supplement: Supplementary file 6 — (A-B) Seahorse extracellular flux analysis demonstrated that intravitreal injection of PPARα-expressing adenovirus restored deficient retinal mitochondrial respiration in 8-week-old Pparα -/- mice (wild-type (WT) ad-GFP n = 19; Pparα -/- ad-GFP n = 25; Pparα -/- ad-PPARα n = 19). (C) Seahorse extracellular flux analysis demonstrated that siRNA knockdown of PPARα in R28 rod precursor cells decreased mitochondrial respiration (siScr n = 7; siPPARα n = 6). (D) siRNA knockdown of PPARα reduces mRNA levels by ~50% in R28 cells (n = 4). (E) NADH oxidation assay revealed that NADH oxidation was decreased in 8-week-old Pparα -/- retinas relative to age-matched WT, which was significantly abrogated by complex I inhibitor rotenone. NADH oxidation was unchanged in Pparα -/- retinas relative to WT in rotenone-inhibited reactions (WT n = 10; Pparα -/- n = 9). B: *P ≤ 0.05 vs. WT-GFP, # P ≤ 0.05 vs. Pparα -/- + GFP, one-way ANOVA with Tukey’s post-hoc comparison. C–E: **P ≤ 0.01; ***P ≤ 0.001; ****P ≤ 0.0001, unpaired two-tailed Student’s t test. Figure S6 pertains to Fig. 5 of the main text. (TIF 12715 kb) [file 12915_2017_451_MOESM6_ESM.tif]

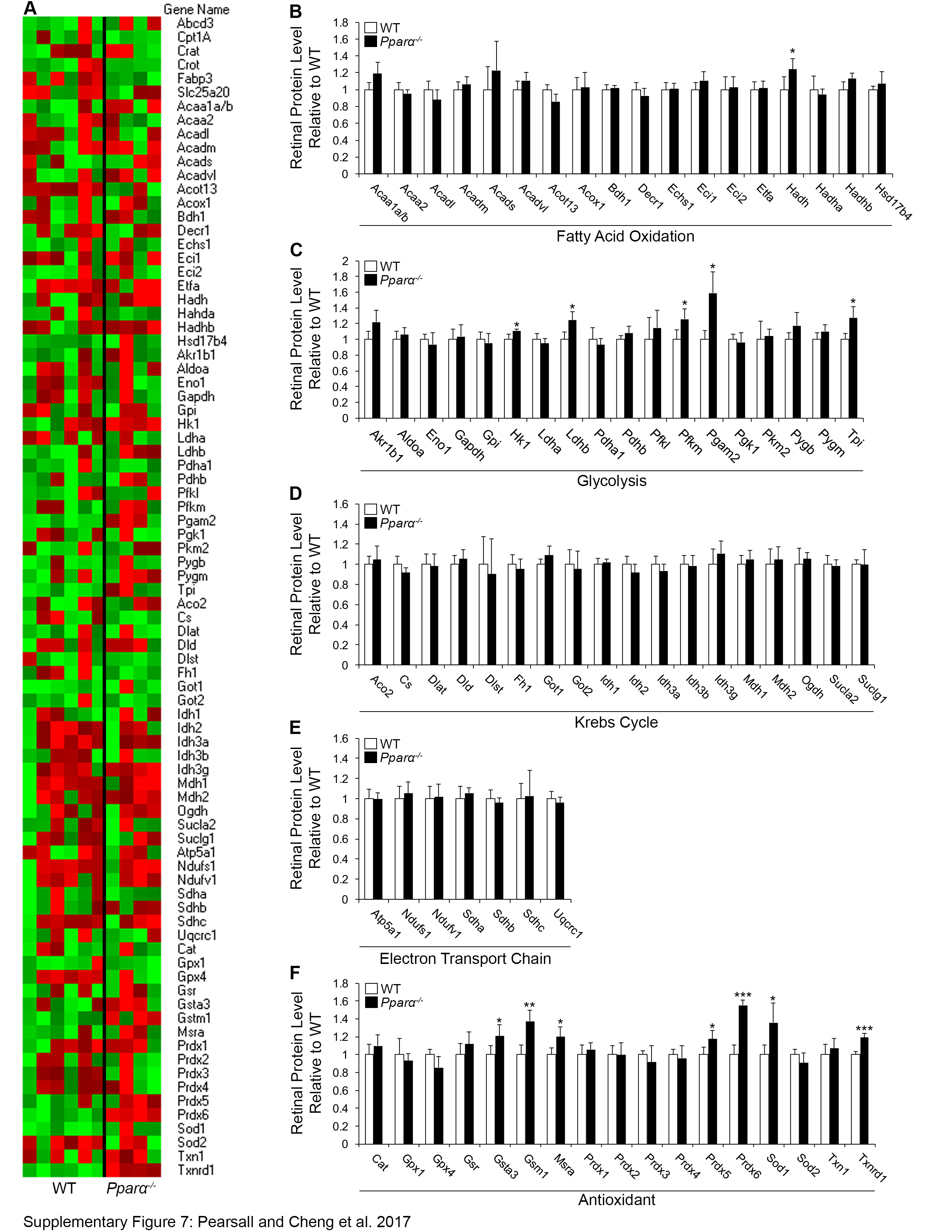

Supplement: Supplementary file 7 — (A) Heatmap representation and gene symbols of proteins measured by proteomic analysis. Please refer to Additional file 10: Table S1 for full gene names. (B) Fatty acid oxidation enzymes are unchanged in Pparα -/- retinas relative to wild-type with exception of Hadha, which is upregulated. (C) Glycolytic enzymes Hk1, Ldhb, Pfkm, Pgam2, and Tpi are upregulated in Pparα -/- retinas. (D) Krebs cycle enzymes are unchanged in Pparα -/- retinas. (E) Electron transport chain enzymes are unchanged in Pparα -/- retinas. (F) Antioxidant enzymes Gsta3, Gsm1, Msra, Prdx5, Prdx6, Sod1, and Txnrd1 are upregulated in Pparα -/- retinas. WT n = 6, Pparα -/- n = 4. *P ≤ 0.05; **P ≤ 0.01; ***P ≤ 0.001, two-tailed unpaired Student’s t test. Figure S7 pertains to Fig. 6 of the main text. (TIF 28635 kb) [file 12915_2017_451_MOESM7_ESM.tif]

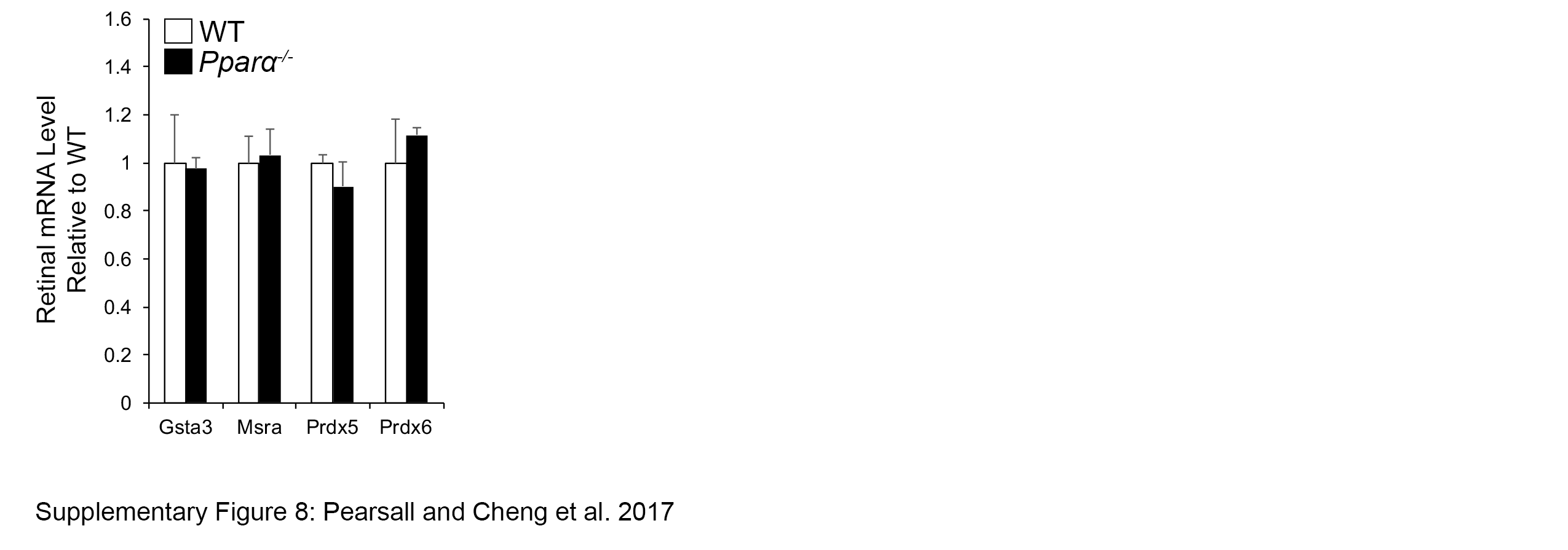

Supplement: Supplementary file 8 — At 4 weeks of age, antioxidant enzymes upregulated at 40 weeks (S7F) are unchanged in Pparα -/- retinas relative to age-matched wild-type. For full gene names refer to Additional file 10: Table S1. n = 5 retinas/genotype. Statistically insignificant as indicated by P ≥ 0.05, two-tailed unpaired Student’s t test. Figure S8 pertains to Fig. 6 of the main text. (TIF 7076 kb) [file 12915_2017_451_MOESM8_ESM.tif]

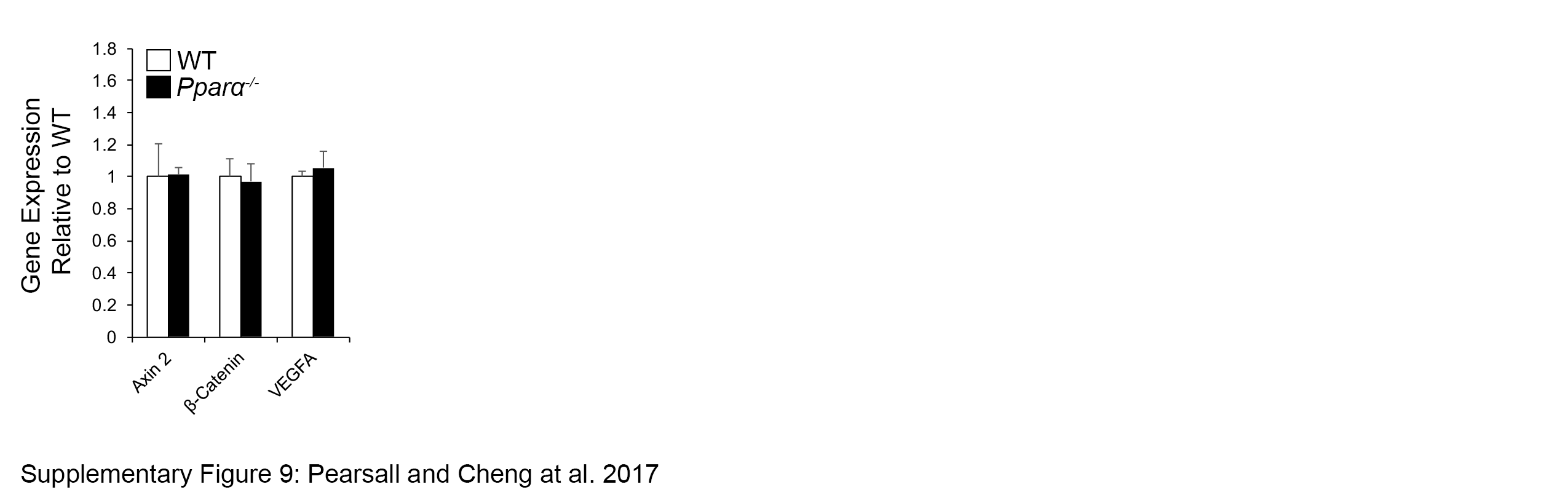

Supplement: Supplementary file 9 — Retinal expression of Wnt target genes was unchanged in Pparα -/- retinas relative to wild-type at 8 weeks of age. N = 5 retinas/genotype. Statistically insignificant as indicated by P ≥ 0.05, two-tailed unpaired Student’s t test. (TIF 6448 kb) [file 12915_2017_451_MOESM9_ESM.tif]
