# Supplementary material for: PPARα is essential for retinal lipid metabolism and neuronal survival
Source: BMC Biol. 2017 Nov 28;15:113. doi: 10.1186/s12915-017-0451-x (PMC5706156; doi:10.1186/s12915-017-0451-x)
Supplement: Supplementary file 10 — Genes presented in Figures S4, S7 and S8. (DOCX 124 kb) [file 12915_2017_451_MOESM10_ESM.docx]

| Additional file 10: Table S1. Genes presented in Figures S4, S7 and S8 | |
| --- | --- |
| Fatty acid oxidation (Fig. S7B, S4E) | |
| Gene Symbol | **Full Name** |
| Acaa1a/b | Acetyl-CoA Acyltransferase 1a/b |
| Acaa2 | Acetyl-CoA Acyltransferase 2 |
| Acadl | Acyl-CoA Dehydrogenase, Long Chain |
| Acadm | Acyl-CoA Dehydrogenase, C-4 to C-12 Straight Chain |
| Acadvl | Acyl-CoA Dehydrogenase, Very Long Chain |
| Acads | Acyl-CoA Dehydrogenase, C-2 to C-3 Short Chain |
| Acot13 | Acyl-CoA Thioesterase 13 |
| Acox1 | Acyl-CoA Oxidase 1, Palmitoyl |
| Bdh1 | 3-Hydroxybutyrate Dehydrogenase, Type 1 |
| Decr1 | 2,4-Dienoyl CoA Reductase 1, Mitochondrial |
| Echs1 | Enoyl-CoA Hydratase, Short Chain, 1, Mitochondrial |
| Eci1 | Enoyl-CoA Isomerase 1 |
| Eci2 | Enoyl-CoA Isomerase 2 |
| Etfa | Electron-Transfer-Flavoprotein, Alpha Polypeptide |
| Hadh | Hydroxyacyl-CoA Dehydrogenase |
| Hadha | Hydratase (Trifuncitonal Protein) Alpha |
| Hadhb | Hydratase (Trifuncitonal Protein) Beta |
| Hsd17b4 | Hydroxysteroid (17-Beta) Dehydrogenase 4 |
| Glycolysis (Fig. S7C) | |
| Gene Symbol | **Full Name** |
| Akr1b1 | Adlo-Keto Reductase Family 1, Member B1 |
| Aldoa | Aldolase A, Fructose-Bisphosphate |
| Eno1 | Enolase 1 (Alpha) |
| Gapdh | Glyceraldehyde-3-Phosphate Dehydrogenase |
| Gpi | Glucose-6-Phosphate Isomerase |
| Ldha | Lactate Dehydrogenase A |
| Ldhb | Lactate Dehydrogenase B |
| Pdha1 | Pyruvate Dehydrogenase (Lipoamide) Alpha 1 |
| Pdhb | Pyruvate Dehydrogeanse (Lipoamide) Beta |
| Pfkl | Phosphofructokinase, Liver |
| Pfkm | Phosphofructokinase, Muscle |
| Pgam2 | Phosphoglycerate Mutase 2 |
| Pgk1 | Phosphoglycerate Kinase 1 |
| Pkm2 | Pyruvate Kinase, Muscle |
| Pygb | Phosphorylase, Glycogen; Brain |
| Pygm | Phosphorylase Glycogen; Muscle |
| Tpi | Triosephosphate Isomerase 1 |
| Krebs Cycle (Fig. S7D) | |
| Gene Symbol | **Full Name** |
| Aco2 | Aconitase 2, Mitochondrial |
| Cs | Citrate Synthase |
| Dlat | Dihydrolipoamide S-Acetyltransferase |
| Dld | Dihydrolipoamide Dehydrogenase |
| Dlst | Dihydrolipoamide S-Succinyltrasferase |
| Fh1 | Fumarate Hydratase 1 |
| Got1 | Glutamic-Oxaloacetic Transaminase 1, Soluble |
| Got2 | Glutamic-Oxaloacetic Transaminase 1, Mitochondrial |
| Idh1 | Isocitrate Dehydrogenase 1 (NADP+), Soluble |
| Idh2 | Isocitrate Dehydrogenase 2 (NADP+), Mitochondrial |
| Idh3a | Isocitrate Dehydrogenase 3 (NAD+), Alpha |
| Idh3b | Isocitrate Dehydrogenase 3 (NAD+), Beta |
| Idh3g | Isocitrate Dehydrogenase 3 (NAD+), Gamma |
| Mdh2 | Malate Dehydrogenase 2, Mitochondrial |
| Ogdh | Oxoglutarate Dehydrogenase (Succinyl-Transferring) |
| Sucla2 | Succinate-CoA Ligase, ADP-Forming, Beta Subunit |
| Suclg1 | Succinate-CoA Ligase, Alpha Subunit |
| Electron transport chain (Fig. S7E) | |
| Gene Symbol | **Full Name** |
| Atp5a1 | ATP Synthase, Alpha Chain, Mitochdondrial |
| Ndufs1 | NADH Dehydrogenase Complex I |
| Ndufv1 | NADH Dehydrogeanse Ubiquinone Complex I |
| Sdha | Succinate Dehydrogenase Complex, Subunit A, Flavoprotein |
| Sdhb | Succinate Dehydrogenase Complex, Subunit B, Iron, Sulfur |
| Schc | Succinate Dehydrogenase Complex, Subunit C, Integral Membrane Protein |
| Uqcrc1 | Ubiquinol-Cytochrome C Reductase Core Protein 1 |
| Antioxidant (Fig. S7F, S8) | |
| Gene Symbol | **Full Name** |
| Cat | Catalase |
| Gpx1 | Glutathione Peroxidase 1 |
| Gpx4 | Glutathione Peroxidase 4 |
| Gsr | Glutathione-Disulfide Reductase |
| Gsta3 | Glutathione-S-Transferase Apha 3 |
| Gstm1 | Glutathione-S-Transferase M1 |
| Msra | Methionine Sulfide Reductase A |
| Prdx1 | Peroxidase 1 |
| Prdx2 | Peroxidase 2 |
| Prdx3 | Peroxidase 3 |
| Prdx4 | Peroxidase 4 |
| Prdx5 | Peroxidase 5 |
| Prdx6 | Peroxidase 6 |
| Sod1 | Superoxide Dismutase 1 |
| Sod2 | Superoxide Dismutase 2 |
| Txn1 | Thioredoxin 1 |
| Txnrd1 | Thioredoxin Reductase 1 |
